# COVID-19 Contact Tracing Strategies During the First Wave of the Pandemic: Systematic Review of Published Studies

**DOI:** 10.2196/42678

**Published:** 2023-06-23

**Authors:** Anna Maria Vincenza Amicosante, Annalisa Rosso, Fabio Bernardini, Elisa Guglielmi, Erica Eugeni, Filippo Da Re, Giovanni Baglio

**Affiliations:** 1 Research and International Relations Unit Italian National Agency for Regional Healthcare Services Rome Italy; 2 Department of Environmental and Prevention Sciences University of Ferrara Ferrara Italy; 3 Regional Directorate of Prevention, Food Safety, Veterinary, Public Health-Veneto Region Venice Italy

**Keywords:** COVID-19, SARS-CoV-2, contact tracing, public health, infectious disease, disease control, community engagement, digital tool

## Abstract

**Background:**

Contact tracing (CT) represented one of the core activities for the prevention and control of COVID-19 in the early phase of the pandemic. Several guidance documents were developed by international public health agencies and national authorities on the organization of COVID-19 CT activities. While most research on CT focused on the use digital tools or relied on modelling techniques to estimate the efficacy of interventions, poor evidence is available on the real-world implementation of CT strategies and on the organizational models adopted during the initial phase of the emergency to set up CT activities.

**Objective:**

We aimed to provide a comprehensive picture of the organizational aspects of CT activities during the first wave of the pandemic through the systematic identification and description of CT strategies used in different settings during the period from March to June 2020.

**Methods:**

A systematic review of published studies describing organizational models of COVID-19 CT strategies developed in real-world settings was conducted in accordance with the PRISMA (Preferred Reporting Items for Systematic Reviews and Meta-Analyses) statement. PubMed, Embase, and Cochrane Library were searched. Studies not providing a description of the organizational aspects of CT strategies and studies reporting or modelling theoretical strategies or focusing on the description of digital technologies’ properties were excluded. Quality of reporting was assessed by using the Template for Intervention Description and Replication Checklist for Population Health and Policy. We developed a narrative synthesis, using a conceptual framework to map the extracted studies broken down by target population.

**Results:**

We retrieved a total of 1638 studies, of which 17 were included in the narrative synthesis; 7 studies targeted the general population and 10 studies described CT activities carried out in specific population subgroups. Our review identified some common elements across studies used to develop CT activities, including decentralization of CT activities, involvement of trained nonpublic health resources (eg, university students or civil servants), use of informatics tools for CT management, interagency collaboration, and community engagement. CT strategies implemented in the workplace envisaged a strong collaboration with occupational health services. Outreach activities were shown to increase CT efficiency in susceptible groups, such as people experiencing homelessness. Data on the effectiveness of CT strategies are scarce, with only few studies reporting on key performance indicators.

**Conclusions:**

Despite the lack of systematically collected data on CT effectiveness, our findings can provide some indication for the future planning and development of CT strategies for infectious disease control, mainly in terms of coordination mechanisms and the use of human and technical resources needed for the rapid development of CT activities. Further research on the organizational models of CT strategies during the COVID-19 pandemic would be required to contribute to a more robust evidence-making process.

## Introduction

### Background

Contact tracing (CT) has historically been one of the key public health response actions to control the outbreak of a novel virus, particularly in the absence of a vaccine [1]. As with other person-to-person infectious diseases, early case detection, identification, and management of contacts through CT was one of the top priorities for interrupting the chain of infection and controlling the spread of SARS-CoV-2 [2,3]. Before effective vaccines against SARS-CoV-2 became available in December 2020, CT was one of the few tools globally applied to prevent the spread of infection, combined with physical isolation of infected persons and their close contacts (the so-called quarantine), social distancing, and the use of protective devices in public places.

Several guidance documents were produced and disseminated by international public health agencies and national authorities on the organization of CT activities for COVID-19 control, including indications on the type of human and technical resources needed for the different steps of CT (case notification, contact identification, information, management, and surveillance of contacts) [3-10]. Reports on CT activities implemented in several countries suggest marked differences in the organizational models adopted in different settings based on the characteristics of the local health systems and structures, as well as the diagnostic and tracking capabilities [8,11]. Although adapting CT strategies according to the local epidemiological situation and available resources has been emphasized [1,12-14], little evidence is available on the actual implementation of CT activities in real-world settings. In fact, most published studies on CT for COVID-19 have focused on the combination of traditional CT and digital technologies and on cost-effectiveness, ethical concerns, and governance issues related to the use of digital tools [15-20]. Several studies, including systematic reviews, were aimed at estimating the effectiveness of CT strategies for SARS-CoV-2 control, but they mainly relied on modeling techniques [15,21], given the difficulties in measuring real-world effectiveness [22].

### Aim of the Study

Further understanding the real-world implementation of CT strategies under different conditions, including measures adopted to scale-up activities, would be relevant to support future planning of CT activities for infectious disease control. Therefore, we conducted a systematic review of the literature to provide a comprehensive picture of the organizational aspects of CT activities during the first wave of the pandemic through the systematic identification and description of CT strategies used in different settings from March to June 2020. We decided to focus on the first wave of the pandemic, when CT represented one of the core public health activities for COVID-19 containment, to describe actions taken for the rapid set up of CT strategies and scale up of resources.

## Methods

### Study Protocol

We conducted a systematic review of studies describing organizational models of CT strategies for the surveillance and control of SARS-CoV-2 infection. This systematic review was registered in PROSPERO (International Prospective Register of Systematic Reviews; CRD42021279172). The review was conducted in accordance with the PRISMA (Preferred Reporting Items for Systematic Reviews and Meta-Analyses) statement [23] and the Template for Intervention Description and Replication Checklist for Population Health and Policy (TIDieR-PHP) [24].

### Literature Search Strategy

A preliminary exploratory search was carried out restricting the research field to systematic reviews and meta-analyses only, which did not return any noteworthy results. Subsequently, a more specific research strategy was developed to identify primary studies, adapted for each database, using both Medical Subject Headings (MeSH) terms and free text keywords in the title and abstract fields. We searched relevant databases including PubMed, Embase and Cochrane Library from January 1, 2020, to July 31, 2021, for published studies in Italian and English with the terms “contact tracing,” “contact investigation,” “case finding,” “case detect*,” “contact examin*,” “contact screen*,” “COVID-19,” “coronavirus,” and “SARS-COV-2,” with no limitations on study design. The complete search strategy is detailed in Multimedia Appendix 1.

### Inclusion and Exclusion Criteria

Studies were included if they provided a description of the organizational aspects of real-world CT strategies (eg, resources involved and activities conducted in each step of the CT process) applied during the first pandemic wave, with no restrictions on study type, setting, or population. The eligibility criteria (Textbox 1) for this review are described according to the PICOS (Population or Problem, Intervention, Comparison, Outcome, and Study Type) framework. Studies that did not provide a description of the organizational aspects of CT strategies or reported or modeled theoretical CT strategies were excluded. Comments, opinions, editorials, and news reports in which no original information was reported were excluded. In the initial screening phase, we classified studies describing CT strategies focusing on digital application tools such as exposure notification, Bluetooth, GPS, or big data management technologies. Because of their peculiarity, studies focusing on the description of the technological features of digital applications were evaluated separately and were therefore excluded from this review.

**Inclusion criteria for this review.**

**Population or problem**

- Organization of contact tracing (CT) activities in populations hit by COVID-19 during the first pandemic wave.

**Intervention**

- Any real-world CT strategies for the control of COVID-19 during first wave not centered on exposure notification, Bluetooth, GPS, or big data management technologies.

**Comparison**

- Either no or any type of real-world CT strategy for the control of COVID-19, depending on whether comparative analyses are available in the included studies.

**Outcome**

- Identification of the main elements characterizing the organization of CT activities for COVID-19 control.
- Description of the main types of CT strategies for COVID-19 control.

**Study type**

- All types of studies.
- Papers published in peer-reviewed journals, in Italian or English, available in full-text.

### Data Extraction and Study Quality

Search results were imported into a reference management database (EndNote 7.8 [Clarivate Plc]). Duplicate articles were removed, and the titles and abstracts of all the collected records were screened by 2 reviewers (AMVA and AR). Studies that clearly did not meet the inclusion criteria were excluded. Full texts of potentially relevant articles were retrieved and independently examined by the 2 researchers. The reference lists of retrieved articles were also searched to identify other potentially relevant studies. All excluded articles and reasons for exclusion were recorded (Multimedia Appendix 2) and any disputes between the 2 researchers were resolved through discussion.

A standardized data extraction file was developed, including the following information: main author, year of publication, country, study design, study period, epidemic phase, type of CT program (institutional level or local level), study population, study setting, activities carried out during the various steps of the CT process (case notification, contact identification, information, management, and surveillance), human and technical resources used, main features of the CT model, and quantitative results.

The quality of reporting was assessed by 2 reviewers (AMVA and AR) using the TIDieR-PHP checklist [24]. The checklist enables clear and comprehensive reporting of population health and policy interventions, providing 11 items to capture pertinent features of these interventions. Adherence to these 11 items was assessed in each of the included studies.

We developed a narrative synthesis using a conceptual framework to map the extracted studies broken down by the target population.

## Results

### Overview

**Figure 1:**
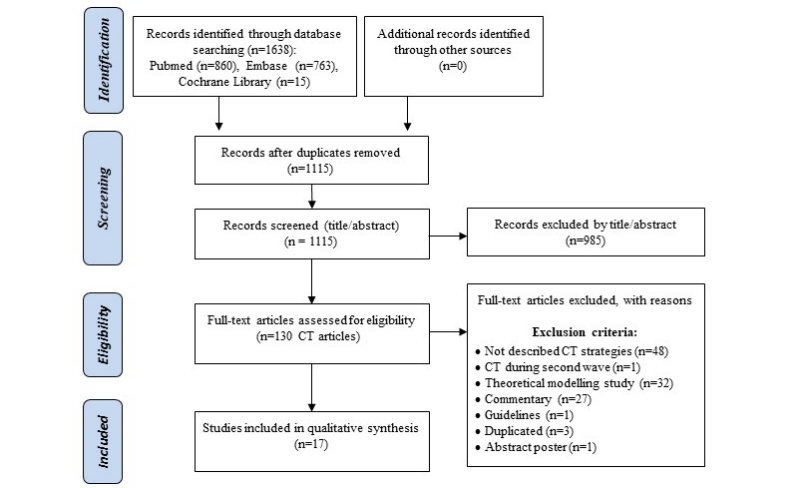
PRISMA (Preferred Reporting Items for Systematic Reviews and Meta-Analyses) flow diagram of included studies [23]. CT: contact tracing.

We retrieved a total of 1638 studies. After duplicate removal and title abstract selection, 130 full texts were assessed, and 17 of them were included in the narrative synthesis [25-41] (Figure 1).

Studies focusing on the organizational models of CT apps and other digital tools were not included in this study and will be addressed in a different review. The main features of the included studies are summarized in Table 1.

All included papers were descriptive accounts of CT strategies implemented in various settings, except for a qualitative study [25]. A total of 8 studies were conducted in the United States [26,27,29-32,36,41], 3 in Asia [34,35,39], 3 in Europe [33,37,40], 2 in Africa [25,28], and 1 in Australia [38].

One study reported information on implemented CT strategies disaggregated for the first and second waves [33], and 1 study did not clearly report the timing when the CT model was implemented [35]. A total of 10 studies described organizational models implemented by national or local governments or public health agencies [25,26,28,29,31,37-41]. The remaining studies described strategies implemented locally in specific contexts [27,30,32-36]. In addition, 7 studies targeted the general population [25-31], and the remaining 10 studies described CT activities carried out in specific population subgroups (workers, travelers, and vulnerable populations) [32-41]. Further details are provided in Table S1 in Multimedia Appendix 3 [25-41].

Quantitative results of CT activities were seldom reported, and the available data were not comparable across the studies (Table S2 in Multimedia Appendix 3).

The results of the assessment of reporting quality are included in Multimedia Appendix 4 [25-41].

**Table 1:** Characteristics of included studies.

Study	Country	Epidemiological scenario	Target	Setting	Institutional level
Asiimwe et al [25], 2021	Africa–Ghana (Greater Accra Region)	First wave	General population	Regional territory	Government
Breeher et al [32], 2020	United States–Minnesota, Florida, and Arizona	First wave	Health care workers and patients	Hospital (Mayo Clinic)	Specific context
Clarke et al [40], 2020	Europe–Ireland	First wave	Detainees	Prison	Government
de Laval et al [37], 2021	Europe–France	First wave	Workers	Working environment (Creil Air Base–MSFAC^a^)	Government
Draper et al [38], 2021	Australia–Northern Territory	First wave	Travelers entering Australia and general population	Airports, ports, and communities	Government
Fields et al [41], 2021	United States–Utah (Salt Lake County)	First wave	General population and PEH^b^	County territory and accommodation for PEH	Government
Hall et al [36], 2021	United States–Virginia	First wave	Workers and their external contacts	Work environment (US Navy Medicine, the Bureau of Medicine and Surgery, Falls Church)	Specific context
Zirbes et al [33], 2021	Europe–Germany	First and second wave	Patients, employees, and health care workers	Marburg University Hospital	Specific context
Kalyanaraman and Fraser [26], 2021	United States–County in Maryland	First wave	General population	County territory	Government
Koetter et al [27], 2021^c^	United States–Central Pennsylvania	First wave	General population, with CT^d^ starting from hospital-diagnosed cases—Penn State College of Medicine	Territory of the region	Specific context
Mak et al [35], 2021	China–Hong Kong	Not clearly stated	Patients	Department of Ophthalmology—United Christian Hospital Hong Kong	Specific context
Mueller et al [28], 2020	Africa–Lagos State	First wave	General population	Territory of 5 subareas with the highest number of cases	Government
Niccolai [29], 2020	United States–Connecticut (New Haven)	First wave	General population and university community	City territory and university (Yale University)	Government
Pelton et al [30], 2021^c^	United States–central Pennsylvania	First wave	General population, with CT starting from hospital-diagnosed cases—Penn State College of Medicine	Territory of the region	Specific context
Quach et al [39], 2021	Vietnam–Hanoi	First wave	Travelers entering Vietnam	Flight	Government
Reid et al [31], 2021	United States–City of San Francisco	First wave	General population	City territory	Government
Wong et al [34], 2020	China–Hong Kong	First wave	Patients and health care workers	Queen Elizabeth Hospital	Specific context

^a^MSFAC: Military Support Facility.

^b^PEH: people experiencing homelessness.

^c^The articles refer to the same study but with 2 different focuses: Koetter et al [27] described the organizational model of contact tracing (CT), whereas Pelton et al [30] focused on key performance indicators.

^d^CT: contact tracing.

### General Population

All studies targeting the general population have described CT models implemented at the local level (regional territories, counties or cities). Two studies focused on cases diagnosed within a university hospital, the Penn State College of Medicine [27,30], while 1 study described CT activities carried out within Yale University’s community, in addition to those targeting the city of New Haven’s general population [29].

Not all studies provided details on case notifications. Where described, case notification envisaged the communication of new COVID-19 cases from test laboratories or test centers to CT teams [27,28,30] or the use of infectious disease notification systems, either with automatic notification [26] or through an active search of new cases conducted by an epidemiologist [29].

All studies reported the identification of close contacts by telephone interviews with cases, except for the model described by Mueller et al [28] in the Lagos area, where contacts were line-listed for possible follow-up at the point of sample collection. A total of 3 studies specified that the interviews with cases were conducted using defined questionnaires or forms [25,27,29] developed by epidemiologists. In all cases, the contact list was entered into an electronic database or software.

Contacts were notified of their exposure to a COVID-19 case via telephone in all models. In 2 cases [26,31], the phone call was accompanied by a letter, email, or SMS text messaging notifying the exposure. Koetter et al [27] reported that the CT team from the Penn State College of Medicine used a premade phone script designed with assistance from epidemiologists treating infectious diseases to provide information on exposure and quarantine.

The management and monitoring of contacts during quarantine (14 days in all studies) was carried out mainly using telephone [25-28]. As an alternative to telephone monitoring, the model implemented in the Lagos area also provides home visits by nurses to assess symptoms and take swabs of symptomatic contacts [28]. The students of Penn State College of Medicine [27,30] monitored the symptoms of the cases contacted through a questionnaire sent by email from the CT management software (REDCap [Research Electronic Data Capture], Vanderbilt University) with automatic feedback to the CT team. In the model described by Reid et al [31], screening of symptoms during quarantine occurred via SMS text messaging sent automatically from the COVID-19 tracking application CommCare (Dimagi Inc) and subsequent feedback to the CT team.

The CT models retrieved from the literature used different types of human resources. In some cases, only health workers were involved [25,26], providing different roles depending on their professional background. In the model implemented in the Accra Region [25], clinicians based at the regional level informed contacts of exposure and supervised the work of community nurses engaged in contact monitoring, whereas case investigations were conducted by epidemiologists and disease control officers at the local level. The model described by Kalyanaraman et al [26] involved CT teams composed of nurses responsible for investigating cases and informing contacts, as well as health assistants and runners responsible for monitoring contacts, under the supervision of an epidemiologist. Some studies have reported the involvement of medical and health profession students in CT activities. At the Penn State College of Medicine, medical students were involved in the CT of cases diagnosed within the hospital, being employed either in the management of cases (case teams) or contacts (contact teams) [27]. Yale University health care professional students were involved on a voluntary basis in a CT program aimed at both campus and off-campus populations in collaboration with the local health department, which provided web-based training for volunteers [29]. The University of California San Francisco also involved medical students in a CT program developed with the San Francisco Department of Public Health, and public health experts from the 2 bodies coordinated the activities and trained the CT team, which also included retired doctors, librarians, and other civil servants [31].

All the models described used software or web platforms for case and contact management. Most studies used open-source platforms (eg, Surveillance Outbreak Response Management and Analysis System, SORMAS Foundation [25], and Open Data Kit, Jekyll and Minimal Mistakes [28]) or relied on existing platforms such as such as RedCap (Vanderblit University) [27,30] or Veoci Inc (Virtual Emergency Operations Center Software) [29]. The CT model developed by the students of University of California San Francisco and the experts from San Francisco Department of Health was initially partnered with Dimagi Inc to make use of their web-based COVID-19 tracking application, CommCare, and then transitioned to the digital platform, CalConnnect, to align San Francisco with most other jurisdictions in California [31].

Only 2 models provided the development of indicators that allowed for the measurement of the effectiveness of the interventions [30,31]. In both cases, the development of a CT model based on the rapid mobilization of human resources (including university students), team organization, and the use of digital platforms led to a reduction in the time required to complete CT activities. The study conducted at the Penn State College of Medicine showed a reduction in the test turnaround time from 21.8 to 2.3 days, also due to improvements in the testing capacity [30]. The study by Reid et al [31] showed a reduction in the time between contact registration and the first attempted contact from 5 days to 1 day over a 2-month period.

### Special Populations

Studies describing CT models not addressed in the general population targeted the following population groups: workers, including health care workers (HCWs), military workers, and civilians working in military areas [32-37]; travelers [38,39]; and vulnerable populations, including inmates and homeless people [40,41]

#### Workers

Among studies carried out on workers, 4 studies focused on CT models developed in the health care setting [32-35] and 2 studies reported CT strategies addressed to civilians and military workers.

##### HCWs and Patients

Studies describing CT models addressing HCWs also reported CT activities in patients and contacts; 1 study focused on hospital patients only [35]. One study was carried out in the Mayo Clinic campuses, United States [32]; 1 was carried out in the University Hospital of Marburg, Germany [33]; and all the other studies were conducted in Hong Kong public health service hospitals.

Where indicated, the structure responsible for notifying cases among HCWs was the Occupational Health Services (OHS) [32] or the Infection Control and Prevention (ICP) service [33]. The procedure for case notification was not described in all studies. In the model described by Wong et al [34], a positive health worker was notified by the hospital’s in-house laboratory, while in the model described by Breeher et al [32], case notification was made by multiple parties (infectious disease control teams, public health bodies, or self-reporting).

Contacts identification was carried out mainly by hospital internal teams: in the model described by Breeher et al [32] it was carried out by a designated exposure triage provider (ETP) composed of doctor and nurses, in collaboration with the OHS [32], while in other models, contact identification was conducted by the ICP service [33,35], sometimes in collaboration with the local health department [35]. Different approaches have been described for contact listing: some models relied on cases filling standard forms available on the web on the hospital intranet [32,33] and telephone interviews [32]; other models relied on information provided by the ward manager [34] or collected through a hospital software tracking the movement of patients within the hospital (UQ Web) [35]. Patients’ contacts were identified through different digital information systems (the electronic medical record, EMR [32]; health information systems [33]; patient administration CT systems [34]; and UQ Web software [35]. All studies classified contacts according to the levels of exposure, risk, and subsequent definition of isolation measures.

Information on contacts was managed internally in all models as well: it was carried out either by hospital staff (eg, by the ETP, the exposure investigation team, and the nursing exposure investigation team in the model described by Breeher et al [32] or by the ICP in the model described by Zirbes et al [33]) or directly by cases using an intranet platform [33].

In the Mayo Clinic model, cases self-filled an internet form and were then interviewed by telephone to assess the level of risk, define isolation measures and prescribe diagnostic tests [32]. In the Marburg Hospital model, the ICP automatically notified HCWs identified as close contacts to define work restrictions and patients to begin isolation through the intranet platform. HCWs were sent to the clinic’s testing centers, and results were available on the intranet, while the patients were cared by the local health authorities [33]. In the Hong Kong model described by Wong et al [34], quarantine for close contacts was arranged at a designated camp (staff) or in isolation rooms for patients with airway-transmitted infections, whereas casual contacts were subjected to medical surveillance.

Management of contacts included monitoring of symptoms, in some cases self-reported by the HCWs [32], and testing. Surveillance of symptoms duration ranged from 14 [33] to 28 [34] days from the last contact with the index case. The Marburg University Hospital model envisaged repeating antigenic tests (for health professionals) and polymerase chain reaction (PCR) tests (for patients) 3 times every 48 hours since the last contact with the index case [33], while Wong et al [34] reported that all contacts were monitored daily for temperature and symptoms for a 28 days (including the quarantine of 14 days).

The human resources used and their functions were not always clearly reported. The Mayo Clinic model detailed the inclusion of different professionals at the central level in Richmond and at the local campuses: at the central level, nurses and doctors—as part of the ETP—conducted case investigation, the exposure investigation team (composed of clinicians reallocated from other departments) conducted contacts’ risk assessment, and administrative or relocated laboratory staff supported data collection; at the local level, nurses exposure investigation team established work restrictions and arranged testing for symptomatic cases [32]. In the model described by Zirbes et al [33], the ICP worker was the key figure in all the CT workflow (in December 2020, 3 ICP workers managed to monitor up to 1201 contacts).

Data on cases and contacts were collected and managed using ad hoc software [32,33,35]. In addition, software solutions were used to track cases and’ movement of contacts within the hospital [35] or to allow cases and contacts to fill in standard forms, for contact listing, collecting information on exposure and symptoms [32,33].

##### Military Area Workers

Two studies addressing CT in the workplace described activities conducted in military areas: 1 at the headquarters of the US Navy Medicine in Virginia [36] and the other at the Creil Air Base in France [37]. Both the studies describe CT activities implemented further to notification of a COVID-19 case within the workplace: in the model described by Hall et al [36], the hospitalized index case self-notified SARS-CoV-2 infection to the office manager, whereas in the French study, the index cases (whose number is not reported) were diagnosed within the military base. In both studies, cases were notified to the local health authorities.

Military personnel [37] or the public health officer (PHO) embedded within the US Navy Medicine headquarters [36] were responsible for investigating the contact, drawing up the contact list, and identifying the possible source of infection. The local health department investigated contacts outside the workplace [36].

Information to contacts was based on their risk level [36,37]. In the study by de Laval [37], all personnel at the air base were classified as close contacts and the model focused on prompt testing of all new symptomatic people. In the US Navy study, the PHO sent information to contacts via mass email with confirmation of receipt, prescribing self-observation, or home quarantine for 14 days for low- and medium-risk contacts, respectively [36]. All personnel were instructed to contact the base health center [37] or their doctor upon onset of symptoms [36]. In the study performed by de Laval et al [37], tests were carried out directly in the base, in a specially set up field-sampling unit.

Surveillance of all identified contacts was performed daily via telephone [36,37], and in case of development of symptoms, PCR testing was required [37].

Human resources used were not specified in the study by de Laval et al [37]. In the US Navy Medicine model, all CT activities within the office were conducted by the PHO, who arranged and coordinated workspace sanitation and was responsible for investigating the case, informing and monitoring contacts, and for campaigning internal information. Administrative staff were responsible for the internal information campaign and infection control policies.

In all studies, CT activities were conducted via telephone, email, and interviews with standardized questionnaires; the PHO also made use of invitation lists, meeting attendance, and carpooling data [36].

#### Travelers

Two studies described CT strategies used for travelers: one focused on an index case identified on a cruise ship arriving in the Northern Territory of Australia [38] and the second focused on a CT of a flight arriving in Vietnam [39].

In both studies, the index cases were confirmed by a PCR test, but the notification procedure was not described.

In the case of a confirmed positive traveler, the identification of contacts started from the passenger list of the same flight or cruise. In the study performed by Draper et al [38], the passenger list was provided by the airline or the Australian Government Department of Health National Incident Room. In Vietnam, the passenger list was provided by the immigration office and the Civil Aviation Administration to the competent Provincial Center for Disease Control. At the provincial level, local health personnel worked with local authorities, social security departments, and local volunteers to contact passengers and identify their contacts [39].

Draper et al [38] specified that information to contacts was provided via telephone using a standard questionnaire by the contact tracer team (CTT), which also collected data on the time, place, and duration of contact and on COVID-19 symptoms [38]. In the study performed by Quach et al [39], local health personnel interviewed, tracked, tested, and arranged quarantine (for 14 days) in centralized structures for primary and secondary contacts or suggested self-quarantine at home [39].

Contact monitoring was performed randomly by compliance officers by sending daily SMS text messages to monitor the development of fever or respiratory symptoms and compliance with quarantine measures [38]. Contacts in centralized quarantine had their symptoms and temperature checked twice daily; swabs were collected after 3 to 5 days and on day 13 before exiting quarantine [39]. Accommodation, meals, and basic hygiene needs were provided by the Ministry of Health. Any contact who tested positive during centralized or home quarantine was transferred to a referral hospital for isolation and monitoring [39]. Both studies did not provide information on the composition of the CT teams.

Tools used for CT included the Telstra Integrated Messaging platform [39] for sending SMS text messages and a web-based epidemiology database (NetEpi) to collect information on close contacts [38].

#### Vulnerable Populations

Two studies described CT strategies in vulnerable populations. One study addressed CT of inmates within the Irish prison system [40], and the study performed by Fields et al [41] described CT strategies of people experiencing homelessness (PEH) hosted in quarantine or isolation facilities in Salt Lake County.

The notification of index cases was only described in the prison context, where prison staff notified the internal CTT of positive cases or symptomatic (experiencing cough and fever) inmates [40].

Contacts were identified through interviews with notified inmates and analysis of closed-circuit television footage [40]. For PEH, case investigations were conducted by dedicated staff visiting isolation and quarantine facilities using a standard form, initially in person and later using prepaid mobile phones or walkie talkies at the facilities.

Only Clarke et al [40] reported details on information to contacts. In the prison system model, the CTT informed inmates’ contacts, arranged for their isolation, provided indications on home self-quarantine to family members and staff, and informed the public health agency.

Inmates’ contacts quarantined inside prisons received clinical monitoring daily, whereas prison staff’s contacts in home quarantine were monitored by the community contact management program; however, casual contacts were instructed to self-monitor in case of any COVID-19 symptoms [40]. The PEH were monitored by nurses who filled out a spreadsheet with medical and epidemiological information for each person housed in quarantine or isolation facilities [41].

The human resources involved in CT were described in the prison model. Each prison had a CTT made up of at least 4 people, including security chiefs, assistant chief officers, prison officers, psychologists, or clerical staff, whereas doctors and nurses were responsible for informing positive results and for clinical monitoring. The involved staff followed a training program developed by the National Infection Control Team, public health agency, and National Quality Improvement team [40]. In PHE facilities, nurses were responsible for collecting information on cases and contacts and for monitoring symptoms, and no information was provided on the profile of contact tracers [41].

All models envisaged the collection of case and contact information in local and sometimes centralized databases. Irish prisons collected information on cases and contacts using an Excel (Microsoft Corporation) spreadsheet and saved data in the penitentiary’s IT system, which was then sent by secure email to the National Infection Control Team and the public health agency to be archived in the Health Service Executive central database [40]. Information on PEH was collected using the existing Utah National Electronic Disease Surveillance System or EpiTrax software [41].

## Discussion

### Principal Findings

A systematic review of the published literature on the organizational models of CT implemented during the first wave of the COVID-19 pandemic identified a limited number of studies. Despite the fact that published literature on the topic is scarce, some elements characterizing the setup of different CT programs and some recommendations to increase the efficacy of CT activities can be drawn.

A common feature of all studies was the decentralization of CT activities at the local level: CT was delegated to regions, counties, metropolitan areas, or specific settings such as hospitals, prisons, and communities, with the involvement of local call centers or human resources, as opposed to a centralized approach where CT is usually conducted in a national or central center [11]. However, in most models all steps of CT (case identification, identification, and monitoring of contacts) were implemented locally; in others, the identification of contacts and the overall management of CT activities took place at a central level [25,32,40]. Centrally managed models were mainly described in specific settings characterized by an internal CT tracing system (eg, the Mayo Clinic model in the study by Breeher et al [32] and the Irish Penitentiary Institutes model described by Clarke et al [40]). The model described by Asiimwe et al [25], implemented in the Great Accra Region of Ghana, followed the structure of the country’s health system, with activities carried out in 3 tiers: national, regional, and district tiers with a strong focus on community care [42]. Evidence from European case studies suggests that the governance and organization of CT systems follow the structure of health systems, with a greater decentralization of activities in countries with regional management of health services [8]. According to the European Centre for Disease Control and Prevention (ECDC), the decentralization of CT systems represents a challenge for the collection of comprehensive and harmonized data on the volume and effectiveness of the interventions carried out that need to be addressed [8].

Several studies have described the involvement of human resources not belonging to public health agencies as contact tracers, such as university students from health faculties [27,29-31], hospital health workers, staff of CT programs implemented within health facilities [32], or representatives of other organizations, such as United Nations agencies (eg, study conducted by Mueller et [28] in Nigeria, with the involvement of the WHO [World Health Organization] and UNFPA [United Nations Population Fund] staff). As the number of cases increased, the main international public health agencies (Centers for Disease Control and Prevention [CDC], ECDC, and WHO) recommended the mobilization of nonpublic health staff, such as students, community health workers, volunteers, and civil servants, provided that they are adequately trained and supervised by public health bodies responsible for epidemic control [3,4,43,44]. Many of the documented experiences complied with this recommendation, showing its effectiveness in reducing the time needed to complete CT activities and in increasing the number of people contacted [27-32].

A common element emerging from all studies is the need to use IT software to support CT activities, as the number of cases and contacts to be monitored increased. The use of digital technology can overcome challenges related to incomplete contact identification, delays in the identification and isolation of cases, and notification and quarantine of contacts. Available evidence has been synthetized in some reviews, suggesting the effectiveness of digital technologies in supporting the control of the epidemic, but also underlying several normative, technical, and acceptance barriers to be addressed [45-47]. The WHO stressed the need to integrate such tools into comprehensive and adequately resourced CT strategies [4].

Different CT models were based on multiagency collaboration, with partnerships between public health agencies and other actors (eg, universities, United Nations agencies, community organizations, companies, and other institutions). Collaboration across different actors was not only aimed at the mobilization of human resources but also at the exchange of information; in the CT models aimed at travelers [38,39], collaboration between public health bodies and airline companies, port or airport management, and flight control bodies was essential to help trace persons who may have been exposed to SARS-CoV-2 during flights, as also recommended by international guidelines [48].

Three studies reported the use of outreach strategies to carry CT activities through home visits to closed settings (eg, prisons, shelters for PHE) [25,38,41]. This approach was used to address susceptible communities, which are difficult to reach through usual communication channels (telephone, email). The outreach approach was also used to reach out to the general population in the 2 models developed in African Countries (Ghana and Nigeria), where community worker programs are widely implemented [25,38]. The positive role of outreach activities conducted by community health workers in improving the effectiveness of CT interventions was previously highlighted in a systematic review conducted in the context of tuberculosis control [43]. The review indicated the potential value of outreach and community health workers in conducting case investigations in specific populations, such as drug addicts or homeless people. For these populations, it also indicated that location-based strategies of CT might lead to identification of an increased number of contacts, as also described in the model developed by Fields et al [41] for PHE.

CT programs aimed at health care professionals were characterized by the direct involvement of in-hospital OHS [32] and ICPs in CT activities [32,33]. In the Marburg University Hospital model described by Zirbes et al [33], ICP workers were the pivot of the CT process, accessing all information related to cases and contacts through a web-based platform. As also suggested by ECDC guidance on infection prevention and control and preparedness for COVID-19 in health care settings, potential mitigation measures, including CT, need to be addressed in collaboration with the existing OHS or health and safety committees [49]. As stressed by Breeher et al [32], CT in hospital settings may be extremely resource intensive. The use of electronic tools and organization into functional teams were shown to have improved efficiency and integration of standardized processes and made CT scale up feasible.

The 2 studies describing CT in the workplace, both conducted in the military setting, were based on an integration of the work conducted by public health agencies and staff responsible for occupational health and safety (in the US Navy model described by Hall et al [36]), with the presence of PHOs embedded in the workplace. CT activities in the workplace and collaboration between the different bodies responsible for safety at work, also by virtue of the existing legislation in various countries, are strongly recommended to limit the spread of the SARS-CoV-2, helping to reduce the need for close work activities [50-52]. International bodies also recommend promptly testing symptomatic workers and providing for their isolation, as described in the strategy developed by de Laval et al [37].

Finally, attention was paid to some of the models for the development of strategies aimed at promoting community engagement, which is recognized as an essential element for the success of CT programs [53]. Two studies have emphasized the importance of addressing the social needs of individuals placed in isolation and quarantine (eg, food, drugs, and connection to other services), also to increase compliance with isolation measures [26,27]. Another element facilitating community engagement was the involvement of contact tracers speaking other languages to ensure compliance of linguistic minorities with isolation and quarantine [27,31].

In general, one of the main findings of our literature review was the scarcity of published studies specifically aimed at describing the organizational aspects of CT activities. This could be because of difficulties in measuring and describing the effectiveness of CT interventions, making the topic of little interest in the scientific community [15]. Conducting a proper evaluation of the effectiveness of CT, testing, and isolation interventions is a complex task, as the type of evidence required is difficult to obtain (ie, randomization of interventions is not ethically acceptable); hence, available evidence is mainly based on modeling techniques or proxy data [2,22,54]. Most published studies on CT have focused on the development of mathematical models aimed at describing the factors determining the effectiveness of CT [55]. Though these studies can be useful in estimating the effectiveness of CT under different assumptions, they cannot provide indications on the organizational aspects of CT activities. Disseminating information on how CT strategies have been developed in different contexts, including data on human and technical resources, organization, coordination, and governance of activities, could provide useful elements for future planning and contribute to “evidence making” in this field. The absence of empirical data during the COVID-19 pandemic has challenged the traditional evidence-based approach, requiring other ways of generating and synthesizing evidence, where narrative studies on CT organizational models represent examples of evidence-making interventions [56,57].

### Limitations

Only a few studies included in our review attempted to provide data on the effectiveness of the intervention, by identifying and calculating some key performance indicators to detect improvements in the effectiveness of CT interventions (mainly measured by a reduction in the time needed to complete CT activities or an increase in the number of cases and contacts reached) following some adjustments in the organization of activities (eg, increase in human resources and adoption of a digital tool). As also suggested in recent systematic reviews on the effectiveness of CT strategies for infectious disease control, more evidence is needed to understand how to optimize the effectiveness of CT across a range of settings and contexts, including large-scale comparative studies [15], informing “how, where, and when” to deploy CT most effectively [15]. Few data collected were not fully comparable across different studies. As suggested by Vogt et al [22], a universally agreed set of indicators is needed to allow for cross-system comparisons and to improve the performance of CT systems.

The main limitation of this systematic review was the focus on the first phase of the epidemic (first wave, March to June 2020) only. Our primary aim was to describe how CT strategies were first developed in different settings to respond to a novel virus outbreak. Nevertheless, collecting and synthesizing information on how CT strategies were adapted to changes in the epidemiological situation would be relevant to support the future planning of CT activities in response to viruses characterized by different modes of transmission, incubation time, and virulence. Furthermore, we did not consider changes that occurred with the availability of COVID-19 vaccines at the end of 2020, when most public health efforts were directed toward the organization of vaccination campaigns, and research mainly focused on measuring vaccine effectiveness.

Therefore, further evidence is needed to evaluate the adaptation of CT models to the different phases of the epidemic, including strategies adopted to scale up interventions, integration of traditional and digital methods, and adjustments of CT with regard to vaccination status.

### Conclusions

In conclusion, our systematic review provides some preliminary evidence on organizational models of CT developed across various settings and contexts during the first wave of the COVID-19 pandemic. We identified some common elements in all strategies that allowed for the effective development of CT activities in the early phase of a novel virus epidemic, including decentralization of activities (case notification, identification, and management of contacts), involvement of nonpublic health trained resources, use of digital tools for CT management, interagency collaboration, adoption of strategies to increase community engagement, and aspects peculiar to each setting (eg, outreach, involvement of OHS and ICPs). Despite the lack of data on CT effectiveness, these findings can provide some indications for future planning and development of CT strategies for infectious disease control. Further research on the organizational models of CT strategies during the COVID-19 pandemic, including data on real-world effectiveness and on strategies developed during the following phases of the epidemic, would be needed to contribute to a more robust evidence-making process.

## Appendix

Multimedia Appendix 1 Search strategy.

Multimedia Appendix 2 List of excluded studies.

Multimedia Appendix 3 Summary of findings of included studies [25-41].

Multimedia Appendix 4 Template for Intervention Description and Replication Checklist for Population Health and Policy items for the quality of studies [25-41].

## Abbreviations

CDC: Centers for Disease Control and Prevention
CT: contact tracing
CTT: contact tracer team
ECDC: European Centre for Disease Control and Prevention
ETP: exposure triage provider
HCW: health care worker
ICP: Infection Control and Prevention
MeSH: Medical Subject Headings
OHS: Occupational Health Services
PCR: polymerase chain reaction
PEH: people experiencing homelessness
PHO: public health officer
PICOS: Population or Problem, Intervention, Comparison, Outcome, and Study Type
PRISMA: Preferred Reporting Items for Systematic Reviews and Meta-Analyses
PROSPERO: International Prospective Register of Systematic Reviews
REDCap: Research Electronic Data Capture
TIDieR-PHP: Template for Intervention Description and Replication Checklist for Population Health and Policy
UNFPA: United Nations Population Fund
WHO: World Health Organization
