# Supplementary material for: COVID-19 Contact Tracing Strategies During the First Wave of the Pandemic: Systematic Review of Published Studies
Source: JMIR Public Health Surveill. 2023 Jun 23;9:e42678. doi: 10.2196/42678 (PMC10337430; doi:10.2196/42678)
Supplement: Multimedia Appendix 1 [file publichealth_v9i1e42678_app1.docx]

**Multimedia Appendix 1 - Search strategy for data base**

**Key words**

**"contact tracing"; “contact investigation”; “Case finding”; “case detect*”; “contact examin*”; “contact screen*” ; covid-19 ;** coronavirus ; SARS-COV-2;

**Filters:** human; italian, english ; 01-01-2020 / 01-07-2021

**PUBMED - Results 860**

#1 “contact tracing” [Title/Abstract] – 3.069

#2 "contact investigation*"[Title/Abstract] - 837

#3 "case finding"[Title/Abstract] – 5.346

#4 "case detect*"[Title/Abstract] – 2.949

#5 "contact examin*"[Title/Abstract] - 109

#6 "contact screen*"[Title/Abstract] - 202

#7 (((((#1) OR (#2)) OR (#3)) OR (#4)) OR (#5)) OR (#6) – 11.740

#8 covid-19[Title/Abstract] – 139.734

#9 coronavirus [Title/Abstract] – 71.120

#10 sars-cov-2 [Title/Abstract] – 51.629

#11 ((#8) OR (#9)) OR (#10) – 164.845

#12 (#7) AND (#11) – 1.473

#13 filters 2020/1/1 -2021/7/1 – 1.372

#14 filters human - 882

**#15 filters english - 860**

**EMBASE results 763**

#13). #7 AND #11 AND [english]/lim AND [humans]/lim AND [embase]/lim AND [2020-2021] /py **763**

#12). #7 AND #11 1,221

#11). #8 OR #9 OR #10 162,654

#10. 'sars cov 2':ti,ab 43,322

#9. coronavirus:ti,ab 63,549

#8. 'covid 19':ti,ab 133,754

#7. #1 OR #2 OR #3 OR #4 OR #5 OR #6 14,626

#6. 'contact screen*':ti,ab 249

#5. 'contact examin*':ti,ab 135

#4. 'case detect*':ti,ab 4,259

#3. 'case finding':ti,ab 6,738

#2. 'contact investigation*':ti,ab 956

#1. 'contact tracing':ti,ab 3,148

**COCHRANE 15**

#1 "contact tracing":ti,ab 63

#2 "contact investigation*":ti,ab 31

#3 "case finding":ti,ab 388

#4 "case detect*":ti,ab 0

#5 "contact examin*":ti,ab 0

#6 "contact screen*":ti,ab 0

#7 #1 OR #2 OR #3 OR #4 OR #5 OR #6 464

#8 covid-19:ti,ab 5886

#9 coronavirus:ti,ab 2337

#10 sars-cov-2:ti,ab 256

#11 #8 OR #9 OR #10 6145

#12 #7 AND #11 with Cochrane Library publication date Jan 2020 and Jul 2021 15
