# Supplementary material for: COVID-19 Contact Tracing Strategies During the First Wave of the Pandemic: Systematic Review of Published Studies
Source: JMIR Public Health Surveill. 2023 Jun 23;9:e42678. doi: 10.2196/42678 (PMC10337430; doi:10.2196/42678)
Supplement: Multimedia Appendix 2 [file publichealth_v9i1e42678_app2.docx]

**Multimedia Appendix 2**

**List of studies read in full text and excluded (with reasons for exclusion)**

| **Studies** | **Reasons for exclusion** |
| --- | --- |
| Contact tracing in the context of COVID-19. Interim guidance. Pediatria i Medycyna Rodzinna. 2020;16(1):33-9. | Guideline |
| Abeler J, Bäcker M, Buermeyer U, Zillessen H. COVID-19 Contact Tracing and Data Protection Can Go Together. JMIR Mhealth Uhealth. 8: ©Johannes Abeler, Matthias Bäcker, Ulf Buermeyer, Hannah Zillessen. Originally published in JMIR mHealth and uHealth (http://mhealth.jmir.org), 20.04.2020.; 2020. p. e19359. | Commentary |
| Abueg MHRWNLLPWWAEPSYRMDMCZNAA-. Modeling the effect of exposure notification and non-pharmaceutical interventions on COVID-19 transmission in Washington state. npj Digital Medicine. 2021;4(1). | Theoretical modelling study |
| Agrawal S. Efficacy of manual and digital contact tracing of COVID-19. Indian Journal of Forensic Medicine and Toxicology. 2021;15(1):499-504. | Commentary |
| Akhmetzhanov ARJSMCHYTRN. A hospital-related outbreak of SARS-CoV-2 associated with variant Epsilon (B.1.429) in Taiwan: transmission potential and outbreak containment under intensified contact tracing, January–February 2021. International Journal of Infectious Diseases. 2021;110:15-20. | Commentary |
| Althoff KN, Coburn SB, Nash D. Contact Tracing: Essential to the Public Health Response and Our Understanding of the Epidemiology of Coronavirus Disease 2019. Clin Infect Dis. 2020;71(8):1960-1. | Commentary |
| Amaku M, Covas DT, Coutinho FAB, Azevedo RS, Massad E. Modelling the impact of contact tracing of symptomatic individuals on the COVID-19 epidemic. Clinics (Sao Paulo). 2021;76:e2639. | Theoretical modelling study |
| An YLSJSPHSYKT. Privacy-oriented technique for COVID-19 contact tracing (PROTECT) using homomorphic encryption: Design and development study. Journal of Medical Internet Research. 2021;23(7). | Theoretical modelling study |
| Asabere NY, Acakpovi A, Ofori EK, Torgby W, Kuuboore M, Lawson G, et al. SARPPIC: Exploiting COVID-19 Contact Tracing Recommendation through Social Awareness. Comput Math Methods Med. 2020;2020:3460130. | Theoretical modelling study |
| Awang H, Yaacob EL, Syed Aluawi SN, Mahmood MF, Hamzah FH, Wahab A, et al. A case-control study of determinants for COVID-19 infection based on contact tracing in Dungun district, Terengganu state of Malaysia. Infect Dis (Lond). 2021;53(3):222-5. | Commentary |
| Baraniuk C. How Northern Ireland relaunched contact tracing for covid-19 a month before the rest of the UK. The BMJ. 2020;369 Article Number(m2373). | No CT strategy |
| Barrat A, Cattuto C, Kivelä M, Lehmann S, Saramäki J. Effect of manual and digital contact tracing on COVID-19 outbreaks: a study on empirical contact data. J R Soc Interface. 2021;18(178):20201000. | Theoretical modelling study |
| Bedard BA, Pettit PA. Local Health Departments Cross-Jurisdictional Partnership for Contact Tracing for COVID-19. J Allied Health. 2020;49(3):228-9. | Commentary |
| Beech BM, Woodard L. Contact Tracing: A Clarion Call for National Training Standards. Ethn Dis. 30: Copyright © 2020, Ethnicity & Disease, Inc.; 2020. p. 437-40. | Commentary |
| Bilinski A, Mostashari F, Salomon JA. Modeling Contact Tracing Strategies for COVID-19 in the Context of Relaxed Physical Distancing Measures. JAMA Netw Open. 2020;3(8):e2019217. | Commentary |
| Blomquist PB, Bolt H, Packer S, Schaefer U, Platt S, Dabrera G, et al. Risk of symptomatic COVID-19 due to aircraft transmission: a retrospective cohort study of contact-traced flights during England's containment phase. Influenza Other Respir Viruses. 2021;15(3):336-44. | No CT strategy |
| Bradshaw WJ, Alley EC, Huggins JH, Lloyd AL, Esvelt KM. Bidirectional contact tracing could dramatically improve COVID-19 control. Nat Commun. 2021;12(1):232. | Theoretical modelling study |
| Braithwaite ICTBMARW. Automated and partly automated contact tracing: a systematic review to inform the control of COVID-19. The Lancet Digital Health. 2020;2(11):e607-e21. | No CT strategy |
| Brown J, Ring K, White J, Mackie NE, Abubakar I, Lipman M. Contact tracing for SARS-CoV-2: what can be learned from other conditions? Clin Med (Lond). 2021;21(2):e132-e6. | Commentary |
| Celentano JSDHMEARM. Mobilizing a COVID-19 contact tracing workforce at warp speed: A framework for successful program implementation. American Journal of Tropical Medicine and Hygiene. 2021;104(5):1616-9. | No CT strategy |
| Chan GTRNBdCPWNMSHGKLLDGSMMSS. Adapting active case-finding for TB during the COVID-19 pandemic in Yogyakarta, Indonesia. Public Health Action. 2021;11(2):41-9. | No CT strategy |
| Charbonnier LR-SJCMDMCACLFAONGJ. Contribution of Serological Rapid Diagnostic Tests to the Strategy of Contact Tracing in Households Following SARS-CoV-2 Infection Diagnosis in Children. Frontiers in Pediatrics. 2021;9 Article Number(638502). | No CT strategy |
| Chen WJ, Yang SY, Chang JC, Cheng WC, Lu TP, Wang YN, et al. Development of a semi-structured, multifaceted, computer-aided questionnaire for outbreak investigation: e-Outbreak Platform. Biomed J. 2020;43(4):318-24. | No CT strategy |
| Chiu WA, Fischer R, Ndeffo-Mbah ML. State-level needs for social distancing and contact tracing to contain COVID-19 in the United States. Nat Hum Behav. 2020;4(10):1080-90. | Theoretical modelling study |
| Clark E, Chiao EY, Amirian ES. Why Contact Tracing Efforts Have Failed to Curb Coronavirus Disease 2019 (COVID-19) Transmission in Much of the United States. Clin Infect Dis. 2021;72(9):e415-e9. | Commentary |
| Currie S. COVID-19 contact tracing using sexual health services in Cumbria. Sex Transm Infect. 2020;96(6):395. | Commentary |
| D. DA, Sinopoli ANAGSCGdMAFAJLRILSKCDIP. Strategies to exiting the COVID-19 lockdown for workplace and school: A scoping review. Safety Science. 2021;134 Article Number(105067). | No CT strategy |
| Drake JM, Dahlin K, Rohani P, Handel A. Five approaches to the suppression of SARS-CoV-2 without intensive social distancing. Proc Biol Sci. 2021;288(1949):20203074. | Theoretical modelling study |
| Fiore VG, DeFelice N, Glicksberg BS, Perl O, Shuster A, Kulkarni K, et al. Containment of COVID-19: Simulating the impact of different policies and testing capacities for contact tracing, testing, and isolation. PLoS One. 2021;16(3):e0247614. | Theoretical modelling study |
| Firth JA, Hellewell J, Klepac P, Kissler S, Kucharski AJ, Spurgin LG. Using a real-world network to model localized COVID-19 control strategies. Nat Med. 2020;26(10):1616-22. | Theoretical modelling study |
| Flaherty EA. School Nursing and Public Health: The Case for School Nurse Investigators and Contact Tracing Monitors of COVID-19 Patients in Massachusetts. NASN Sch Nurse. 2020;35(6):327-31. | No CT strategy |
| Fyles M, Fearon E, Overton C, Wingfield T, Medley GF, Hall I, et al. Using a household-structured branching process to analyse contact tracing in the SARS-CoV-2 pandemic. Philos Trans R Soc Lond B Biol Sci. 2021;376(1829):20200267. | Theoretical modelling study |
| Gardner BJ, Kilpatrick AM. Contact tracing efficiency, transmission heterogeneity, and accelerating COVID-19 epidemics. PLoS Comput Biol. 2021;17(6):e1009122. | Theoretical modelling study |
| Giordano G, Blanchini F, Bruno R, Colaneri P, Di Filippo A, Di Matteo A, et al. Modelling the COVID-19 epidemic and implementation of population-wide interventions in Italy. Nat Med. 2020;26(6):855-60. | Theoretical modelling study |
| Girum TLKGMMBSS. Global strategies and effectiveness for COVID-19 prevention through contact tracing, screening, quarantine, and isolation: a systematic review. Tropical Medicine and Health. 2020;48(1). | No CT strategy |
| Goldberg LA, Jorritsma J, Komjáthy J, Lapinskas J. Increasing efficacy of contact-tracing applications by user referrals and stricter quarantining. PLoS One. 2021;16(5):e0250435. | Theoretical modelling study |
| Grantz KH, Lee EC, D'Agostino McGowan L, Lee KH, Metcalf CJE, Gurley ES, et al. Maximizing and evaluating the impact of test-trace-isolate programs: A modeling study. PLoS Med. 2021;18(4):e1003585. | Theoretical modelling study |
| H TNZ, Shu Y, Feng T, Thor Addresses School of Public Health SY-sUSCSCfDC, Control PSCCATFSCfD, Date PSCEfcAIPEDFRE, et al. TLE How Shenzhen, China avoided widespread community transmission: A potential model for successful prevention and control of COVID-19. | Duplicate |
| Hong SH, Hwang H, Park MH. Effect of COVID-19 Non-Pharmaceutical Interventions and the Implications for Human Rights. Int J Environ Res Public Health. 2020;18(1). | No CT strategy |
| Hopewell PC, Reichman LB, Castro KG. Parallels and Mutual Lessons in Tuberculosis and COVID-19 Transmission, Prevention, and Control. Emerg Infect Dis. 2021;27(3):681-6. | Commentary |
| Hu YGJLGLXLXZYCLKYJXSXXGZL. Role of efficient testing and contact tracing in mitigating the COVID-19 pandemic: A network modelling study. BMJ Open. 2021;11(7). | Theoretical modelling study |
| Ish PASGADGN. Contact tracing: Unearthing key epidemiological features of COVID-19. SAGE Open Medical Case Reports. 2020;8. | No CT strategy |
| J TNZ, Sterr CM, Steller M, Dapper L, Nonnenmacher-Winter C, Günther F, et al. TLE Development of a web-based contact tracing and point-of-care-testing workflow for SARS-CoV-2 at a German University Hospital. | Duplicato |
| Jaca A, Iwu CJ, Wiysonge CS. Cochrane corner: digital contact tracing technologies in epidemics. Pan Afr Med J. 2020;37(Suppl 1):8. | Commentary |
| James A, Plank MJ, Hendy S, Binny R, Lustig A, Steyn N, et al. Successful contact tracing systems for COVID-19 rely on effective quarantine and isolation. PLoS One. 2021;16(6):e0252499. | Theoretical modelling study |
| Jung J, Hong MJ, Kim EO, Lee J, Kim MN, Kim SH. Investigation of a nosocomial outbreak of coronavirus disease 2019 in a paediatric ward in South Korea: successful control by early detection and extensive contact tracing with testing. Clin Microbiol Infect. 2020;26(11):1574-5. | Commentary |
| Kant R, Zaman K, Shankar P, Yadav R. A preliminary study on contact tracing & transmission chain in a cluster of 17 cases of severe acute respiratory syndrome coronavirus 2 infection in Basti, Uttar Pradesh, India. Indian J Med Res. 2020;152(1 & 2):95-9. | No CT strategy |
| Kapa S, Halamka J, Raskar R. Contact Tracing to Manage COVID-19 Spread-Balancing Personal Privacy and Public Health. Mayo Clin Proc. 2020;95(7):1320-2. | Commentary |
| Keeling MJ, Hollingsworth TD, Read JM. Efficacy of contact tracing for the containment of the 2019 novel coronavirus (COVID-19). J Epidemiol Community Health. 2020;74(10):861-6. | Theoretical modelling study |
| Kendall M, Milsom L, Abeler-Dörner L, Wymant C, Ferretti L, Briers M, et al. Epidemiological changes on the Isle of Wight after the launch of the NHS Test and Trace programme: a preliminary analysis. Lancet Digit Health. 2020;2(12):e658-e66. | Theoretical modelling study |
| Kim W, Lee H, Chung YD. Safe contact tracing for COVID-19: A method without privacy breach using functional encryption techniques based-on spatio-temporal trajectory data. PLoS One. 2020;15(12):e0242758. | Theoretical modelling study |
| Klaaren J, Breckenridge K, Cachalia F, Fonn S, Veller M. South Africa's COVID-19 Tracing Database: Risks and rewards of which doctors should be aware. S Afr Med J. 2020;110(7):617-20. | No CT strategy |
| Koizumi N, Siddique AB, Andalibi A. Assessment of SARS-CoV-2 transmission among attendees of live concert events in Japan using contact-tracing data. J Travel Med. 272020. | No CT strategy |
| Kucharski AJ, Klepac P, Conlan AJK, Kissler SM, Tang ML, Fry H, et al. Effectiveness of isolation, testing, contact tracing, and physical distancing on reducing transmission of SARS-CoV-2 in different settings: a mathematical modelling study. Lancet Infect Dis. 2020;20(10):1151-60. | Theoretical modelling study |
| Lash RR, Donovan CV, Fleischauer AT, Moore ZS, Harris G, Hayes S, et al. COVID-19 Contact Tracing in Two Counties - North Carolina, June-July 2020. MMWR Morb Mortal Wkly Rep. 2020;69(38):1360-3. | No CT strategy |
| Lash RR, Moonan PK, Byers BL, Bonacci RA, Bonner KE, Donahue M, et al. COVID-19 Case Investigation and Contact Tracing in the US, 2020. JAMA Netw Open. 2021;4(6):e2115850. | No CT strategy |
| Lewis D. Why many countries failed at COVID contact-tracing - but some got it right. Nature. 2020;588(7838):384-7. | Commentary |
| Linden M, Dehning J, Mohr SB, Mohring J, Meyer-Hermann M, Pigeot I, et al. Case Numbers Beyond Contact Tracing Capacity Are Endangering the Containment of COVID-19. Dtsch Arztebl Int. 2020;117(46):790-1. | Commentary |
| Lucas TCD, Davis EL, Ayabina D, Borlase A, Crellen T, Pi L, et al. Engagement and adherence trade-offs for SARS-CoV-2 contact tracing. Philos Trans R Soc Lond B Biol Sci. 2021;376(1829):20200270. | Theoretical modelling study |
| Luo C, Ma Y, Jiang P, Zhang T, Yin F. The construction and visualization of the transmission networks for COVID-19: A potential solution for contact tracing and assessments of epidemics. Sci Rep. 2021;11(1):8605. | Theoretical modelling study |
| Marcel SCALRNSSEHJFMZGSMBAWSIEM. COVID-19 epidemic in Switzerland: On the importance of testing, contact tracing and isolation. Swiss Medical Weekly. 2020;150(11 12). | Commentary |
| Markus I, Steffen G, Lachmann R, Marquis A, Schneider T, Tomczyk S, et al. COVID-19: cross-border contact tracing in Germany, February to April 2020. Euro Surveill. 2021;26(10). | No CT strategy |
| Mathevet IOKTLZKRV. Accounting for health inequities in the design of contact tracing interventions: A rapid review. International Journal of Infectious Diseases. 2021;106:65-70. | No CT strategy |
| Matthias J, Charboneau T, Schaffer C, Rusten J, Whitmer S, de la Paz J, et al. Notes from the Field: COVID-19 Case Investigation and Contact Tracing Program - Spirit Lake Tribe, North Dakota, September-November 2020. MMWR Morb Mortal Wkly Rep. 2021;70(14):533-4. | CT during second wave |
| McAloon CG, Wall P, Griffin J, Casey M, Barber A, Codd M, et al. Estimation of the serial interval and proportion of pre-symptomatic transmission events of COVID- 19 in Ireland using contact tracing data. BMC Public Health. 2021;21(1):805. | Theoretical modelling study |
| Megnin-Viggars OCPM-TGJWDRGJ. Facilitators and barriers to engagement with contact tracing during infectious disease outbreaks: A rapid review of the evidence. PLoS ONE. 2020;15(10 October). | No CT strategy |
| Mettler SKPJÖOMLKHPHRHCMMH. The importance of timely contact tracing — A simulation study. International Journal of Infectious Diseases. 2021;108:309-19. | Theoretical modelling study |
| Moon SA, Scoglio CM. Contact tracing evaluation for COVID-19 transmission in the different movement levels of a rural college town in the USA. Sci Rep. 2021;11(1):4891. | Theoretical modelling study |
| Ng Y, Li Z, Chua YX, Chaw WL, Zhao Z, Er B, et al. Evaluation of the Effectiveness of Surveillance and Containment Measures for the First 100 Patients with COVID-19 in Singapore - January 2-February 29, 2020. MMWR Morb Mortal Wkly Rep. 2020;69(11):307-11. | No CT strategy |
| Nguyen TA, Cuong QN, Kim ALT, Huong TN, Nguyen HN, Fox GJ, et al. Adapting a TB contact investigation strategy for COVID-19. Int J Tuberc Lung Dis. 2020;24(5):548-50. | Commentary |
| Ortiz NVELJTBKLRJNSSLWCMBAEdSJ. Epidemiologic findings from case investigations and contact tracing for first 200 cases of coronavirus disease, santa Clara County, California, USA. Emerging Infectious Diseases. 2021;27(5):1301-8. | No CT strategy |
| Panovska-Griffiths J, Kerr CC, Stuart RM, Mistry D, Klein DJ, Viner RM, et al. Determining the optimal strategy for reopening schools, the impact of test and trace interventions, and the risk of occurrence of a second COVID-19 epidemic wave in the UK: a modelling study. Lancet Child Adolesc Health. 2020;4(11):817-27. | Theoretical modelling study |
| Park OPYJPSYKYMKJLJPEKDJBHRB. Contact transmission of Covid-19 in South Korea: Novel investigation techniques for tracing contacts. Osong Public Health and Research Perspectives. 2020(1):60-3. | No CT strategy |
| Park YJ, Choe YJ, Park O, Park SY, Kim YM, Kim J, et al. Contact Tracing during Coronavirus Disease Outbreak, South Korea, 2020. Emerg Infect Dis. 2020;26(10):2465-8. | No CT strategy |
| Perry BL. Contact Tracing Could Exacerbate COVID-19 Health Disparities: The Role of Economic Precarity and Stigma. Am J Public Health. 1112021. p. 778-81. | No CT strategy |
| Persaud PSJSBKE-MASB. Effective contact tracing strategies for COVID-19: A municipal health department's model. Open Forum Infectious Diseases. 2020;7(SUPPL 1):S165. | No CT strategy |
| Persichilli J. COVID-19 Testing and Contact Tracing in New Jersey. MD Advis. 2020;13(2):30-1. | Commentary |
| Pham TQ, Hoang NA, Quach HL, Nguyen KC, Colquhoun S, Lambert S, et al. Timeliness of contact tracing among flight passengers during the COVID-19 epidemic in Vietnam. BMC Infect Dis. 2021;21(1):393. | No CT strategy |
| Phillips L, Knobbe KR, Carson BN, Jorgensen L, Truong S, Mitchell J, et al. Lessons from Contact Tracing in Mid-Missouri. Mo Med. 2021;118(1):81-4. | No CT strategy |
| Pilny A, Joseph HC. An egocentric network contact tracing experiment: testing different procedures to elicit contacts and places. International journal of environmental research and public health [Internet]. 2021; 18(4):[1‐11 pp.]. Available from: https://www.cochranelibrary.com/central/doi/10.1002/central/CN-02247484/full. | No CT strategy |
| Pogreba Brown K, Austhof E, Rosa Hernández AM, McFadden C, Boyd K, Sharma J, et al. Training and Incorporating Students in SARS-CoV-2 Case Investigations and Contact Tracing. Public Health Rep. 2021;136(2):154-60. | No CT strategy |
| Pokharel A, Soulé R, Silberschatz A. A case for location based contact tracing. Health Care Manag Sci. 2021;24(2):420-38. | No CT strategy |
| Pung R, Chiew CJ, Young BE, Chin S, Chen MI, Clapham HE, et al. Investigation of three clusters of COVID-19 in Singapore: implications for surveillance and response measures. Lancet. 2020;395(10229):1039-46. | No CT strategy |
| Ready T. Covid-19: The US state copying a global health template for contact tracing success. The BMJ. 2020;369 Article Number(m1890). | Commentary |
| Reintjes R. Lessons in contact tracing from Germany: Germany built on existing local infrastructure to get ahead of the covid-19 pandemic. The BMJ. 2020;369 Article Number(m2522). | Commentary |
| Rich SN, Richards VL, Klann EM. Factors Contributing to Missing COVID-19 Cases During Contact Tracing. JAMA Intern Med. 2021;181(7):1014-5. | Commentary |
| Ross AM, Zerden LS, Ruth BJ, Zelnick J, Cederbaum J. Contact Tracing: An Opportunity for Social Work to Lead. Soc Work Public Health. 2020;35(7):533-45. | No CT strategy |
| Ruebush E, Fraser MR, Poulin A, Allen M, Lane JT, Blumenstock JS. COVID-19 Case Investigation and Contact Tracing: Early Lessons Learned and Future Opportunities. J Public Health Manag Pract. 2021;27 Suppl 1, COVID-19 and Public Health: Looking Back, Moving Forward:S87-s97. | No CT strategy |
| Sachdev DD, Brosnan HK, Reid MJA, Kirian M, Cohen SE, Nguyen TQ, et al. Outcomes of Contact Tracing in San Francisco, California-Test and Trace During Shelter-in-Place. JAMA Intern Med. 2021;181(3):381-3. | Commentary |
| Sachdev DD, Cohen SE, Scheer S. Factors Contributing to Missing COVID-19 Cases During Contact Tracing-Reply. JAMA Intern Med. 2021;181(7):1015. | Commentary |
| Schneider J, Love W, Rusie L, Flores A, Tadesse B, Hazra A, et al. COVID-19 Contact Tracing Conundrums: Insights From the Front Lines. Am J Public Health. 2021;111(5):917-22. | No CT strategy |
| Sciamanna CN, Du P, Cialdini RB. Using Persuasion science to improve COVID-19 contact tracing. Am J Infect Control. 2021;49(4):528-9. | No CT strategy |
| Sen-Crowe B, McKenney M, Elkbuli A. Utilizing technology as a method of contact tracing and surveillance to minimize the risk of contracting COVID-19 infection. Am J Emerg Med. 2021;45:519. | No CT strategy |
| Shelby T, Hennein R, Schenck C, Clark K, Meyer AJ, Goodwin J, et al. Implementation of a volunteer contact tracing program for COVID-19 in the United States: A qualitative focus group study. PLoS One. 2021;16(5):e0251033. | Described in another study |
| Smathers SDRLGLLDG-mCTJCSESJS. Patient outcomes of contact tracing for COVID-19 in a pediatric hospital. Open Forum Infectious Diseases. 2020;7(SUPPL 1):S309-S10. | Abstract poster |
| Sobers NPHCHJSMGNSHHMMMQKHIR. Impact of COVID-19 contact tracing on human resources for health – A Caribbean perspective. Preventive Medicine Reports. 2021;22 Article Number(101367). | Theoretical modelling study |
| Squeri R, Levita A, Intelisano R, Costa GB, Mancuso G, Grasso L, et al. Correct management and low rate of contagiousness of healthcare workers in a University Hospital in Southern Italy: from contact tracing to serological investigation. Acta Biomed. 2020;91(9-s):79-86. | No CT strategy |
| Steinbrook R. Contact Tracing, Testing, and Control of COVID-19 - Learning from Taiwan. JAMA Internal Medicine. 2020;180(9):1163-4. | No CT strategy |
| Sun K, Viboud C. Impact of contact tracing on SARS-CoV-2 transmission. Lancet Infect Dis. 2020;20(8):876-7. | Commentary |
| Terebuh PD, Egwiekhor AJ, Gullett HL, Fakolade AO, Miracle JE, Ganesh PT, et al. Characterization of community-wide transmission of SARS-CoV-2 in congregate living settings and local public health-coordinated response during the initial phase of the COVID-19 pandemic. Influenza Other Respir Viruses. 2021;15(4):439-45. | No CT strategy |
| Thai PQ, Rabaa MA, Luong DH, Tan DQ, Quang TD, Quach HL, et al. The First 100 Days of Severe Acute Respiratory Syndrome Coronavirus 2 (SARS-CoV-2) Control in Vietnam. Clin Infect Dis. 2021;72(9):e334-e42. | No CT strategy |
| Torneri A, Libin P, Vanderlocht J, Vandamme AM, Neyts J, Hens N. A prospect on the use of antiviral drugs to control local outbreaks of COVID-19. BMC Med. 2020;18(1):191. | No CT strategy |
| Travis SA, Best AA, Bochniak KS, Dunteman ND, Fellinger J, Folkert PD, et al. Providing a Safe, In-Person, Residential College Experience During the COVID-19 Pandemic. Front Public Health. 2021;9:672344. | No CT strategy |
| Usman AB, Ayinde O, Akinyode A, Gbolahan A, Bello B. Epidemiology of coronavirus disease 2019 (COVD-19) outbreak cases in Oyo State South West Nigeria, March-April 2020. Pan Afr Med J. 2020;35(Suppl 2):88. | No CT strategy |
| Valent F, Gallo T, Mazzolini E, Pipan C, Sartor A, Merelli M, et al. A cluster of COVID-19 cases in a small Italian town: a successful example of contact tracing and swab collection. Clin Microbiol Infect. 2020;26(8):1112-4. | No CT strategy |
| Vallès X, Roure S, Valerio L, López-Muñoz I, Pérez-Quílez O, Soldevila L, et al. SARS-CoV-2 contact tracing among disadvantaged populations during epidemic intervals should be a priority strategy: results from a pilot experiment in Barcelona. Public Health. 2021;195:132-4. | No CT strategy |
| Vecino-Ortiz AI, Villanueva Congote J, Zapata Bedoya S, Cucunuba ZM. Impact of contact tracing on COVID-19 mortality: An impact evaluation using surveillance data from Colombia. PLoS One. 2021;16(3):e0246987. | Theoretical modelling study |
| Wilson A, Warrier A, Rathish B. Contact tracing: a lesson from the Nipah virus in the time of COVID-19. Trop Doct. 2020;50(3):174-5. | Commentary |
| Wu J, Huang Y, Tu C, Bi C, Chen Z, Luo L, et al. Household Transmission of SARS-CoV-2, Zhuhai, China, 2020. Clin Infect Dis. 2020;71(16):2099-108. | Theoretical modelling study |
| Xu D. Modeling of network based digital contact tracing and testing strategies, including the pre-exposure notification system, for the COVID-19 pandemic. Math Biosci. 2021;338:108645. | Theoretical modelling study |
| Yalaman A, Basbug G, Elgin C, Galvani AP. Cross-country evidence on the association between contact tracing and COVID-19 case fatality rates. Sci Rep. 2021;11(1):2145. | Theoretical modelling study |
| Zhang H, Hong C, Zheng Q, Zhou P, Zhu Y, Zhang Z, et al. A multi-family cluster of COVID-19 associated with asymptomatic and pre-symptomatic transmission in Jixi City, Heilongjiang, China, 2020. Emerg Microbes Infect. 92020. p. 2509-14. | No CT strategy |
| Zou H, Shu Y, Feng T. How Shenzhen, China avoided widespread community transmission: a potential model for successful prevention and control of COVID-19. Infect Dis Poverty. 92020. p. 89. | No CT strategy |
