# Supplementary material for: COVID-19 Contact Tracing Strategies During the First Wave of the Pandemic: Systematic Review of Published Studies
Source: JMIR Public Health Surveill. 2023 Jun 23;9:e42678. doi: 10.2196/42678 (PMC10337430; doi:10.2196/42678)
Supplement: Multimedia Appendix 3 [file publichealth_v9i1e42678_app3.docx]

**Multimedia Appendix 3**

**Table S1 - Summary of findings**

| **First author (year)** | **Population** | **Setting** | **Contact tracing process** | | | | **Resources** | | **Results** | **Quantitative results** |
| --- | --- | --- | --- | --- | --- | --- | --- | --- | --- | --- |
|  |  |  |  |  |  |  |  |  |  |  |
|  |  |  | **Case notification** | **Identification of contacts** | **Information to contacts** | **Contacts surveillance** | **Human resources** | **Tools** |  |  |
|  |  |  |  |  |  |  |  |  |  |  |
| **GENERAL POPULATION** | | | | | | | | | | |
| Asiimwe  (2021)  [25] | General population | Accra Region territory | Not described | Identification through forms, collection of information on travel and access to health facilities (for any health reason). | First contact by a supervisor (regional level), who defines the surveillance mode (telephone or home visits). | Assignment of a group of contacts to each community nurse (contact tracer), who performs surveillance according to two strategies: home visits or remote telephone surveillance (according to contact preference), with completion of symptoms diary. On the first home visit, a test is also taken. | Community nurses (community level): contact tracing and telephone or home contact surveillance (2 tracers per visit);  Epidemiologists and disease control officers (local level): contact tracing;  Clinicians (regional level): contacts information and community nurse supervision | Phone  Contact collection sheet  Software: Surveillance Outbreak Response Management- SORMAS and ARC GIS | Local CT model, with the identification of cases and contacts at regional level and the use of contact tracers at community level for contact surveillance. | NR |
| Kalyanaraman  (2021)  [26] | General population | Territory of a county in Maryland | Automatic communication from the National Electronic Disease Surveillance System (NEDSS).  A case file is created and then patients and contacts are assigned to teams. | Assignment of investigations by the team leader.  Telephone contact with the case and updating of close contacts list. | Telephone contact by a nurse. If telephone contact is impossible, delivery of a letter by hand or sending an e-mail. | Daily telephone calls from health assistants to patients and their contacts. | Nurses (3-5 per team): contact information, one nurse is team leader;  Health assistants (1-2 per team) and runners (1-2 per team): contact tracing;  Epidemiologist: supervision of the contact tracing team, daily meetings with the team leader for activity planning. | Computers  Case assignment files and contacts to the team  Telephone  Hand-delivered letters | Case management and contact tracing model by one team.  Management of isolation needs (housing, food, supplies, medication, and connection to other clinical services) by nurses.  Sufficient health resources (early phase of outbreak) for paper-based CT management. | 10 March-30 April 2020: 2024 in the general population (e.g. no nursing homes) 1399 positive cases were identified (of which 1119 were contacted within 24 hours) and a total of 4175 contacts. 3371 contacts were interviewed and quarantined. 390/4175 became cases after testing. |
| Koetter  (2020)  [27] | General population (cases diagnosed in a hospital) | Regional territory (Central Pennsylvania) | From the Penn State College of Medicine (PSCOM) laboratory | Telephone interview by medical students (case team) using a standard form developed by an epidemiologist.  Input of the contact list into the existing Research Electronic Data Capture (REDCap) software. | Telephone contact by medical students (contact team) with case contacts entered in REDCap. Use of a standard form (developed by epidemiologists) to provide information on isolation and detect symptoms. If symptomatic, referral to case team and referral to internal medicine doctor for testing. | Follow up for 14 days following exposure by telephone contact or questionnaire for symptoms detection sent by e-mail by REDCap (automatic feedback to Contact Team).  If a contact becomes symptomatic: notification to internist for scheduling tests and sending to case team for contact tracing. | PSCOM medical students (suspended from clinical internship activities due to COVID risk) specially trained (PSCOM Response Team), organised into "Case Teams" for case management and "Contact Teams" for contact management (120 students involved).  PSCOM internal medicine doctor for performing tests. | Standard forms for epidemiological survey and communication to contacts.  REDCap software for contact list entry and automatic dispatch of symptoms detection questionnaire. | Establishment of a working group for the CT of cases diagnosed by the PSCOM laboratory composed of medical students, who also contributed to the design of the model, and trained new arrivals and supervised activities.  Separation into two teams, one dedicated to case management and the second to contact management.  Utilisation of native students of other languages to overcome communication barriers.  Network with facilities for social support of people in isolation.  Definition of Key Performance Indicators (KPIs) to measure the effectiveness of the model. | March 24 - May 28: 536 confirmed cases identified 953 contacts. Of the 953 contacts 261 with symptoms were treated as presuntive cases |
| Mueller  (2020 )  [28] | General population | Lagos State Territory- 5 sub-areas with the highest number of cases | Notification from COVID-19 testing centres to the local government (LGA) CT team where the case resides.  Active search for cases in health facilities and the community.  Health facilities: examination of patient records for suspicious symptoms by LGA CT team, contact with identified patients and follow-up. Supervision by Medical Officer of Health.  Communities: daily visits to families by 5/10 two-person teams (one volunteer and one community mobiliser) in each area, to identify suspicious symptoms, contact with confirmed cases and travel histories, with support from the State Primary Health Care Council and United Nations Population Fund (UNFPA). Supervision by a health educator.  Once a suspected case is identified for testing, the team informs the local CT team. | Collection of contacts list at the point of sample collection for COVID-19. | The CT team in the LGA reaches out to the line-listed contacts by phone. | The contacts are called daily and information on their signs and symptoms are collected on a contact tracing form for 14 days.  If there is an onset of symptoms, the case investigation team moves to the house of contact that is now a suspected case for sample collection and line-listing of contacts. | Contact tracing teams:  Medical Officers of Health  Disease Surveillance and Notification Officers  Health Educators  National Centre for Disease Control (NCDC) staff  UNFPA and WHO support  Teams of volunteers and community mobilisers for active case finding in the community. | Open data kit (ODK): open source tool that can also be used off-line  Contact tracing form | Decentralisation of contact tracing to the levels of LGA and its branches (ward), with the support of external partners (WHO, UNFPA).  The use of ODK has further facilitated the flow of information. | A total of 4123 confirmed cases;  2886/4123 provided a contact list  4336 contacts identified. No cases among those who have completed quarantine and for others ongoing follow-up. |
| Niccolai  (2020)  [29] | General population and university community | City territory and University (Yale University) | Two CT programmes  1) University: it is not specified who notifies the case to volunteer case interviewers.  2) Health Department: the city epidemiologist identifies COVID-19 positive residents in the electronic infectious disease surveillance database and refers them to the volunteer case interview team. | 1) University: telephone interviews of cases. Information is stored in a database created and managed by Yale Health (respecting data security)  2) Health Department: telephone interviews, with a tool created by the Health Department to guide the interviews; volunteer case interviewers assess any social needs and provide linkages to available supports. | Telephone call to contacts by volunteers, who inform about exposure and give information on testing; self-monitoring of symptoms, link to available resources for assistance. | Unspecified | Volunteers recruited from “health science” schools (primarily students of public health, medicine, and nursing) at Yale University (nearly 200 volunteers recruited via e-mail, nearly 50 volunteers active on a weekly basis).  Online training by state and local health department staff experienced in CT on COVID-19 biology and contact tracing. Case interviewers also trained on interview conduct (instructions, scripts and answers to frequently asked questions). | E-mail and GroupMe (a mobile chat-group app) to communicate between volunteers.  Veoci, a locally based emergency management software platform for case interviews within the Health Department.  Protected health information from both programs was maintained on secure servers managed by the university or city and was shared as needed with volunteers via Yale Secure Box. | Use of volunteers (“health science” students) coordinated by Yale University, in close collaboration with government and local health departments, to implement CT programmes for COVID-19 within the University and for the local population.  The model enabled a strengthening of academic and public health partnerships. | The study describes the first three months of programme development and implementation. At the time of publication, no evaluation of either process or results has been carried out. |
| Pelton  (2021)  [30] | General population (cases diagnosed in a hospital) | Regional territory (Central Pennsylvania) | see Koetter |  |  |  | Involvement of medical and health professional students as contact tracers of cases diagnosed by the University Hospital laboratory: 150 part-time operators involved, equating to approximately 36 full-time operators. | see Koetter | Key performance indicators: - contact-to-case ratio - % percentage of contacts testing positive - time between test execution and receipt of report (test turnaround time)  - time between exposure and insertion of contacts in Research ElectronicData Capture identification number database (RCID) - time between symptoms onset/positive test and first call to cases and contacts (time to initial call)  exposure awareness (% of contacts informed of being contacts) Operations:  - length of call - % of unanswered calls  Interventions: - no. and type of interventions requested from CT staff e.g. letters for absence from work, food needs. | Identified an average of 2.6 contacts per case;  Reached 94% of cases and 84% of contacts by telephone (higher than other programmes reporting 50-60%);  Test turnaround time (time to receive test results) average 5.2, decreasing from 21.8 days to 2.3 days in 11 weeks: partly due to improved testing capabilities;  Average time between symptoms onset and call to contacts: 10.6 days;  261 suspected cases identified, of which only 72 were tested, 52% positive (higher than the national average, 22%)  From March 24 to May 28, the team called 1489 individuals. 536 were PCR confirmed cases of COVID-19. 953 were contacts exposed to people with COVID-19, of which 261 were treated as presumptive cases. |
| Reid  (2021)  [31] | General population | City territory (San Francisco) | Unspecified.  Department Operations Centre (DOC) Testing Branch increased testing capacity for all COVID case contacts, especially in closed communities. | Telephone contact with identified cases, use of a web-based digital platform to compile the contact list for each case. | Telephone contact with contacts, followed by notification via SMS or e-mail via web-based platform.  Testing to all close contacts. | Sending of SMS for symptoms screeening to contacts via web platform for 14 days.  Support during quarantine (food, hygiene, medication) for all San Francisco residents, hotel transfers for those unable to quarantine at home. | Case Investigation and Contact Tracing (CICT), composed of public health experts from San Francisco Department of Public Health-SFDPH and University of California San Francisco (UCSF).  SFDPH staff dedicated to CT and retired medical students and physicians, city and county librarians (CCSF) and other public servants trained by UCSF/SFDPH.  Training on Zoom® platform on: disease transmission (basic information), principles of case isolation and quarantine for contacts, ethics of public health data collection, importance of cultural sensitivity, specifics of local processes and data collection, and characteristics of the San Francisco healthcare system. | CommCare: web-based monitoring application for COVID-19 developed by Dimagi (Boston, Massachusetts)  Later, transition to the CalConnnect platform (Richmond, California). | CT programme characterised by university support, rapid mobilisation of the workforce, effective training and timely introduction of a digital platform for case/contact management.  The platform allowed for the stratification of CT process interventions by language preference and race.  Involvement of Community-Based Organisations (CBOs) to ensure community responsiveness in CT activities.  Adoption of indicators to measure impact:  - no. and % of new cases interviewed,  - no. and % of new cases with at least one contact provided,  - no. and % of cases with at least one contact reached, no. and % of new cases with at least one tested contact,  - no. and % of cases with at least one contact diagnosed with COVID-19. | 13 April - 18 June 2020: 1633 laboratory-confirmed cases (not outbreaks). The SFDPH investigation team reached 1394 of them, traced 1214 close contacts, notified 1017 and tested 457.  During the study period, the number of staff involved increased from 19 (April) to 118 (June); and the time between identification and first contact went from 5 (April) to 1 (June) day. |
| **WORKERS** | | | | | | | | | | |
| Breeher  (2020)  [32] | Healthcare workers | Hospital (Mayo Clinic) | Notification of Covid-positive health worker to the Occupational Health Service (OHS) by local public health partners, IPAC (Occupational Health and Infection Prevention and Control) team or by self-reporting on the part of the infected worker. | Physicians and nurses at central level (Exposure Triage Provider-ETP) identify contacts and assess exposure: creation of a contact list (contact log) by consulting the electronic medical record (EMR) (in the case of patients) or by telephone interview or filling in an electronic form (in the case of healthcare workers), in cooperation with local Occupational Health Service (OHS). Staff from a non-clinical call centre (NCCC) assist the workers in filling in the form. | Central level physicians and nurses (ETP) notify contacts by sending a form to be filled in with information on exposure and symptoms.  A central clinical team (Exposure Investigation Team - EIT) interviews contacts by telephone to assess exposure risk and define restrictive measures (e.g. work suspension) or prescribe diagnostic tests, in collaboration with local nursing teams (Nursing Exposure Team - NET). | Workers fill in an electronic self-assessment form, with support from the call centre (NCCC). If symptoms are detected, the local nursing team (NET) arranges for the test to be performed. | ETP (central level): physicians and nurses specialised in occupational medicine, assess each new case to determine infectious period and identify contacts.  NCCC (central level): administrative or laboratory staff reallocated to assist with data collection.  EIT (central level): clinicians redeployed from the medical department, conduct risk assessment based on the Exposure Assessment Form.  NET (local level): nurses to support Covid-positive workers, establish work restrictions and arrange for testing of symptomatic individuals. | Electronical Medical Record (EMR)  Occupational Case Management (OCM) software: used by the OHS, the case is entered and a notification of exposure sent to contacts.  COVID-19 Positive Self-Assessment Form: Covid-positive cases enter information on symptoms and period of exposure.  COVID-19 Exposure Contact Log: cases enter their contacts, it is supplemented by HR managers.  Exposed Employee Assessment Form: contacts enter information on exposure and symptoms. | Organisational model for in-house CT at the Mayo Clinic, based on the collaboration between a centrally based team (Rochester) consisting of ETP, EIT and NCCC, and the OHS and nursing team locally based at one of the 3 Mayo Clinic campuses. Information collection is mainly computerised. The team works 24 h/7 and the system makes it possible for several operators to work on the same case and contacts in parallel. It represents the evolution of an initial model based solely on EMR consultation and case interviews by the OHS. | NR |
| Zirbes  (2021)  [33] | Healthcare workers and patients | University hospital (Marburg University Hospital) | Not explicitly stated  The healthcare worker index case (the identification mode is not described) is assigned to the ICP (non c'è l'acronimo sciolto Infection Control Prevention???) who schedules the activity with a supervisor or the head nurse via an intranet and e-mail communication; later the hospital's Point-of-care-testing (POCT) information is accessible directly via the intranet. | Initially, the division of infection control (ICP?) was provided with handwritten lists of telephone numbers belonging to healthcare workers who probably had contact with detected index cases. Using the intranet, healthcare workers self-complete a 'contact form' online. The ‘contact form’ generates and updates the tracing list of hospital staff contacts and an anonymous barcode for each contact.  With another online form, healthcare workers report non-hospital contacts to be tested.  The ICP receives automatic notification of high-risk contacts. All other contacts are classified according to the Robert-Koch-Institut (RKI) risk scale adapted to the hospital context.  Contacts among patients are traced separately via HIS and reported to the local health authorities. | In the first phase, the ICP telephones the reported contacts, who are advised to get a PCR-test (on day 1 and day 5), they are told to preventively self-isolate at home and wait for any quarantine orders by the local health authorities, and are asked about any other contacts.  With the automatic notification of high-risk contacts, the ICP gives instructions to isolate the contacts if they are patients or to detach them if they are hospital workers.  With the bar code, the contact accesses the test centre to perform the test. The results of the rapid test are automatically sent via the intranet and visible in the contact list while the PCR results must be reviewed manually by the ICP. | In the first phase, the ICP uses the laboratory information system (LIS) to search for and verify positives. Contact patients are identified separately using the hospital information system (HIS).  With the intranet, healthcare workers, using the barcode, have access to the rapid (antigenic) test at POCT three times every 48 hours since the last contact with the index case; patients and symptomatic contacts are tested in the same scheme with PCR. Each rapid test positive must be confirmed with PCR.  Rapid test results are automatically posted on the intranet and displayed in the contact tracing list. PCR results are reviewed manually. Results are reviewed by the case ICPs on a regular basis. The case ICP has access (password protected) to the contact tracing data for 14 days after entry. | The ICP is the reference figure in the integrated workflow with the POCT. It was estimated that, in December 2020, they were able to handle up to 1,201 contacts with only 3 ICPs on duty. | Intranet for access to formats and information.  Standard format for self-reporting hospital contacts: index case, duration of contact, distance maintained, PPE worn, personal data and existing symptoms.  Format for reporting non-hospital contacts for testing.  RKI scale, adapted to hospital context, for risk classification. | Results: test results obtained quickly, structured and comprehensive contact tracing for hospital employees; the intranet tool provided the ICP with all relevant information at any time by directly linking POCT results to a reference person (ICPs), providing information on the index case and individual risk assessment.  Features: Web-based contact tracing and POCT workflow. Facilitates rapid identification of positive contacts among employees, continuous assessment of the ongoing infection process and assessment of the efficacy of outbreak control measures. | Only anonymised data on the number of contacts, duration of contact, time of contact and date of test results were used for the retrospective analysis of the POCT- ICP workflow effectivity.  Period May-September 2020, 21 index cases; 595 contacts identified among healthcare workers; contact tracing revealed 5 new cases among contacts.  Period October 2020-January 2021: 187 index cases; 3232 contacts identified among healthcare workers; contact tracing revealed 24 news cases among contacts. |
| Wong  (2020)  [34] | Patients and healthcare workers | Hospital (Queen Elisabeth Hospital) | A patient, with symptoms of pneumonia, diagnosed positive for COVID-19 with testing performed in the hospital laboratory. The patient had been in an open ward cubicle with 10 other patients for 35 h before being transferred to the airborne infection isolation room (AIIR). | Tracing of contacts prior to diagnosis.  Patient contacts were identified through the Patient Administration Contact Tracing System, while staff contacts were identified through ward managers.  Hospital staff contacts were interviewed and risk categorized according to the nature of activities, duration of exposure, personal protective equipment (PPE) worn at the time of exposure.  Patients who shared the same cubicle with the index case were considered as ‘patient close contact’. | See contact surveillance column | Close contacts:  • staff close contacts were subjected to a 14-day work exclusion and quarantined at a designated camp site, followed by medical surveillance for another 14 days.  • patient close contacts were quarantined into an AIIR (or quarantine camp if the patient was deemed clinically stable to be discharged from hospital) for 14 days, followed by medical surveillance for 14 days.  All other cases (‘casual contacts’) were subjected to medical surveillance for 28 days with no restriction to work or discharge from hospital.  Body temperature and respiratory symptoms were monitored daily throughout the 28-day period. Any abnormalities were reported to the medical personnel at the quarantine camp, or to the hospital infection control team, with hospitalization into an AIIR and testing of SARS-CoV-2 where indicated. | Not explicitly stated | Not explicitly stated | Study with a small sample size (one index patient), describes contact tracing, nature of exposure and PPE worn and reports transmission dynamics in a hospital ward. | Contact tracing identified: 71 staff contacts (including 7 close contacts) and 49 patient contacts (including 10 close contacts)  Thirty staff and 22 patient contacts developed fever and/or reported respiratory symptoms during the surveillance period; 76 (of 52) respiratory samples sent for RT-PCR for SARS-CoV-2 were collected, all of which were negative. The remaining contacts were asymptomatic during the surveillance period. |
| Mak  (2021)  [35] | Patients and healthcare workers | Hospital (Department of Ophtalmology- United Christian Hospital Hong Kong) | Not explicitly stated | The hospital software (UQ Web) tracks patient movement between different consultation rooms and allows tracing medical personnal who have come into contact with COVID-19 cases. The system generates a list of patients who attended the same consultation room.  In cases of doubt, the Chief Infection Control Officer and/or the Centre of Health Protection makes the exposure risk assessment. | Patients are informed and asked to report onset of symptoms. | Inpatients are monitored for onset of symptoms by the Hospital Infection Control Team (our IOC).  Outpatients are monitored by the Centre for Health Protection. | Not explicitly stated | UQ Web software | Use of the hospital's in-house software, UQ Web, which tracks the movement of patients between different consultation rooms and thus allows the tracing of staff and other patients who have come into contact with COVID-19 cases. | The system has been used for tracing patients three times, with approximately 40 patients traced. |
| Hall  (2020)  [36] | Workers and their external contacts | Working environment with military and civilian workers (Headquarters for U.S. Navy Medicine, the Bureau of Medicine and Surgery in Falls Church) | The index case, undergoing hospital treatment, notifies the office manager who in turn informs the public health officers (PHO) in the headquarters office | The PHO calls the index case requesting:  permission to interview him/her; information on travel in the last few months, interaction with infected persons or from places where COVID-19 outbreaks were reported, symptoms (cough and fever), work activities carried out in the 3 days after the onset of symptoms (work meetings with internal and external colleagues, national and international conferences).  Identifies/compiles the contacts list. | The PHO consults the local health department conducting the investigation for contacts outside the workplace.  The PHO informs contacts in the office according to their risk classification:  - low risk: information via a mass e-mail with confirmed receipt and instructions for self-observation for 14 days;  - medium risk: they were invited to remain at home under quarantine conditions for 14 days after the last day of exposure;  - high risk: no cases.  All contacts were instructed to contact their doctor if symptoms developed. | The PHO did daily follow-up of medium-risk contacts who were self-monitoring Covid-19 symptoms | The office manager requests the PHO to initiate the CT.  The PHO arranges and co-ordinates the sanitisation of the workspaces and those used by the staff (bathrooms, printing rooms, communal lounges ...) and informs the contacts and carries out their follow-up.  Administrative offices reorganise work (increased telework) and, with the assistance of the PHO, office management distributed informative messages, held virtual meetings and evaluated infection control policies.  Local public health authorities managed the tracking of contacts outside work. | Telephone and e-mail.  The PHO used lists of invitations and attendance at meetings to identify contacts, and the PHO also identified potential contacts of workers who had used the carpool (passenger list request) with the index case. | In the event of a workplace outbreak, rapid and coordinated responses between public health experts and office managers can be effective in controlling the spread of an infectious outbreak. Characteristic of the CT model is the presence of the PHO in the office. | 150 contacts identified of which 10 developed Covid-like symptoms resulting in isolation, self-monitoring and awaiting PCR laboratory test result (all negative). |
| de Laval  (2021)  [37] | Workers (military and civilian personnel) | Military Air Base (Creil Air Base - MSFAC) | Index case diagnosed by military service and notified to regional health authorities | Military personnel, immediately after notification of the index case, begin the epidemiological investigation with a search for contacts and possible origin of the infection. | How contacts are notified is not specified.  All base personnel are considered to be close contacts: they are asked to notify the base health officer to perform a test at the onset of symptoms. The test is carried out directly at the base, in a specially established 'field sampling unit'.  Suspect cases excluded following 2 negative PCR tests. | All confirmed cases are isolated for 14 days, medically monitored on a daily basis (at home or in hospital) according to the severity of symptoms, individual risk of worsening and proximity to frail individuals. Symptomatic personnel are asked to perform a PCR test at the base hospital.  Self-monitoring? | It is not clearly stated who does what and when. | Epidemiological survey: telephone interviews using a standardised questionnaire. | Despite the lack of contingent resources (masks, testing), the adoption of appropriate and well-adapted measures (area decontamination, government guidance and CT) interrupted viral transmission within a few days. All cases in MSFAC were linked in a single chain of transmission.  A rigorous investigation strategy based on targeted and systematic testing of symptomatic and at-risk patients, with isolation of cases and at-risk contacts, immediately stopped the spread of the COVID-19 cluster. | From February 25 to March 4, 2020, 24 cases were confirmed: 14 with RT-PCR and 10 with serology; 3 totally asymptomatic.  All RT-PCR confirmed cases and 323 contact persons were interviewed; 119 of whom had real at-risk contacts and were therefore quarantined (14 days). |
| **TRAVELLERS** | | | | | | | | | | |
| Draper  (2020)  [38] | Travellers and contacts with the general population | Cruise ship and destination territory (Northern Territory of Australia) | Case confirmed positive with RT-PCR; not described how notification is made.  Focus on cases in travellers (cruise ship) entering the Northern Territory of Australia. | In the case of a confirmed positive traveller, the company or the Australian Government Department of Health National Incident Room (NIR) provides the passenger manifest. | A team of contact tracers telephones all identified contacts and collects information on the time, place, duration of the contact, the presence of COVID-19 symptoms, and to inform them that they have been quarantined (14 days from the last contact with the positive) at home or in a hotel and that the contact will have to respond daily with a SMS about his/her health status and adherence to the quarantine. | Compliance officers conduct random visits to contacts in quarantine to check adherence to the quarantine requirements of the regulations. | Neither the relevant organisation nor the composition of the 'contact tracers team' nor of the 'compliance officers' is made explicit | NetEpi® online epidemiological database available to jurisdictional public health units.  Telephone, SMS using Telstra Integrated Messaging | The study focused on transmission of COVID-19 to household and close contacts from a cruise ship with known transmission on board. The Australian public health strategy is to do rapid detection/testing/isolation of cases and suspects.  Relevant to this CT strategy is the ability to increase the availability of human resources for deployment. | Proportion of monitored contacts becoming cases (CI 95%)  445 contacts monitored of which 80 developed symptoms and 4 were positive:  - 2 positive/46 close contacts (cruise ship) monitored (4.3%; 95% CI 0.5-14.8%);  - 2 positive/51 household close contacts monitored (3.9%; 95% CI 0.5-13.5%). |
| Quach  (2021)  [39] | Travellers | Air flight (VN54 arriving in Vietnam) | The passenger index case was identified 4 days after arrival in Vietnam (PCR-confirmed positive). It is not explicit who notifies the case.  The COVID case is defined according to the guidelines (LG) of the Ministry of Health of Vietnam. | Classified as:  - primary contacts: all passengers on the flight;  - secondary contacts: close contacts with primary contacts between arrival in Vietnam and the beginning of the isolation/quarantine;  - third generation contacts: non-close contacts with primary/secondary contacts between arrival in Vietnam and the beginning of the isolation/quarantine. The Ministry of Health and the National Steering Committee for COVID-19 Prevention and Control (NSCPC) are tasked with intensive contact tracing of all flight-related primary and secondary contacts. The flight manifest was obtained from the Vietnam Immigration Bureau and the Civil Aviation Administration and distributed to the relevant provincial Center of Disease Control for contact tracing.  At provincial levels, local health staffs cooperates with local government authorities, social security department and local volunteers in order to locate and contact passengers and identify their contacts. | All primary contacts are interviewed to collect information regarding their secondary and third generation contacts, tested and transferred to mandatory (14 days) quarantine at centralized facilities.  Secondary contacts are interviewed, tested and systematically quarantined (for 14 days) in centralized facilities (like primary contacts).  For third generation contacts, the local health staff asks them to quarantine at home (14 days). | Persons in centralised quarantine: are monitored twice a day for symptoms and temperature; they are tested at the beginning of quarantine, after 3-5 days and on the 13th day before the end of quarantine. Accommodation, meals and basic hygiene necessities are provided free of charge by the Ministry of Health.  Level 3 contacts: are monitored daily by health personnel for symptoms; become secondary contacts if their primary or secondary contact becomes positive (tested and transferred to centralised quarantine).  Any person positive or with COVID-19 symptoms, traced by local health staffs or at any point during centralised or home quarantine, is immediately transferred to a reference hospital for isolation and monitoring. | Central level: not explicit.  Provincial level: health staff (composition not made explicit). | All interviews are conducted using a standard questionnaire. | Outbreak with limited secondary cases: CT model based on intensive tracking, sensitive testing and strict mandatory passenger quarantine.  Multi-agency collaboration, sensitive testing policy and strict quarantine mechanisms. | Contacts: 16 flight operators and 201 passengers.  - Primary contacts: 167 passengers and 16 operators were successfully tracked. 15 confirmed cases + index case.  - Secondary contacts: 1000 identified and quarantined;  - Third level contacts: 311 identified and quarantined |
| **VULNERABLE POPULATIONS** | | | | | | | | | | |
| Clarke  (2020)  [40] | Detainees | Prison | Notification to the Contact Tracer Team (CTT) by prison staff of confirmed or highly probable cases (cough and fever) | The CTT identifies close and casual contacts through interviews and analysis of CCTV footage. | The CTT informs close and casual contacts, orders the isolation of detainees and close contacts, gives instructions on home self-quarantine to family members and staff, and informs the Public Health Agency (PH). | Detainees: quarantine within the prison and daily clinical monitoring.  Staff: quarantine at home, reporting to PH and monitoring by community Contact Management Programme (CMP).  Casual contacts: self-monitoring of any Covid symptoms. | Contact Tracer Teams (CTTs) for each prison consisting of at least 4 persons from among:  - Security Chiefs;  - Assistant Chief Officers;  - Prison Officers;  - Assistant psychologist;  - Clerical staff.  Resources trained with a training package agreed with National Infection Control Team (NICT), PH and National Quality Improvement (QI).  Doctors and nurses: report positivity to COVID-19, conduct clinical monitoring of contacts. | The case and contact information (close/casual) is reported in an Excel® sheet and stored in the prison IT system.  The information is sent by secure e-mail to the NICT and PH which stores it in a central database of the Health Service Executive (HSE). | Contact management programme - CMP in prisons, defined with national public health agencies (NICT; PH; QI).  Contact tracing activities are carried out by adequately trained in-prison personnel (CTT) (158 trained operators throughout the country). | There were no confirmed cases during the study period and therefore no conclusions on effectiveness can be drawn. |
| Fields  (2021)  [41] | People experiencing homelessness (PEH) and general population (GP) | PEH isolation/quarantine accommodation and county territory (Salt Lake County) | Not reported. | PEH population: a dedicated staff visits cases and contacts in isolation/quarantine facilities, carries out the epidemiological survey with a standard form. Initially the interviews are in-person, later via pre-paid mobile phones or walkie-talkies.  Visits are carried out daily, if the person to be interviewed is not present up to 4 attempts are made.  Nursing staff compile a spreadsheet with medical and epidemiological information for each person housed in the quarantine/isolation facilities, from entry to exit.  PG: CT conducted by telephone by Salt Lake Health Department staff on all persons tested positive for COVID-19. | Not reported. | Not reported. | Nursing staff to collect data on facility guests; profile of contact tracers unspecified. | Data on all cases and contacts were entered into the existing Utah National Electronic Disease Surveillance System (UT-NEDSS or EpiTrax) software. | CT carried out at PEH isolation facilities, either in person or by using mobile phones/walkie talkies.  Difficulties in identifying and reaching PEH persons (unreachable, lost to follow-up during isolation). Contact tracing for PEH persons should not only be 'person-based' but also 'location-based', collecting information on where persons were at the time of diagnosis and in the previous 48 hours (as recommended by CDC). | PEH 169 laboratory test positive  23/169 hospitalised  2/169 dead  65/127 (information available) with symptoms  93 interviewed, 73 not traced, 3 refused to participate  24 lost to follow-up in quarantine  50 respondents identified through interviews, of which 31 tested  GP 163 laboratory test positive  23/163 hospitalised  2/163 dead  162/163 with symptoms  163 interviewed  0 lost to follow-up in quarantine  758 respondents identified through interviews, of which 322 tested |

**Table S2 - Quantitative results**

| **Study** | **Period** | **Index cases** | **N. contacts identified** | **N. of cases among contacts** |
| --- | --- | --- | --- | --- |
|  |  |  |  |  |
| **General population** | | | | |
| Asiimwe et al [25] | 12 March- 27 June 2020 | NR | NR | NR |
| Kalyanaraman et al [26] | 10 March -30 April 2020 | 1399  (of which 1119 contacted within 24 h) | 4175  (3371 contacts were interviewed and quarantined) | 390 |
| Koetter et al [27] | 24 March – 28 May 2020 | NR | 953 (contacts of COVID-19 case) | 536 confirmed case  261 presumptive cases |
| Muller et al [28] | until 29 May | 4123  2886/4123 provided the contact list | 4336 | 0 among those who have completed quarantine  ongoing follow-up for others |
| Niccolai et al [29] | 14 March – 25 May 2020 | 1117 | 1024 contacts notified | NR |
| Pelton et al [30] | 24 Mar – 28 May 2020 | NR | 1489 | 536 confirmed case  261 presumptive cases |
| Reid et al [31] | 13 April - 18 June 2020 | 1633 (of which 1394 contacted) | 1214 close contacts identified  1017 notified  457 tested | NR |
| **Healthcare workers and patients** | | | | |
| Breeher et al [32] |  | NR | NR | NR |
| Zirbes et al [33] | May-September 2020  October 2020 – January 2021 | Workers: 21  Patients: NR  Workers: 187  Patients: NR | Workers: 595  Patients: NR  Workers: 3232  Patients: NR | Workers: 5  Patients: NR  Workers: 24  Patients: NR |
| Wong et al [34] | February 2020 | Workers: NR  Patients: NR | Workers: 71  (of which 7 close)  Patients: 49  (of which 10 close) | Workers: 0  Patients: 0 |
| Mak et al [35] | NR | Workers: NR  Patients: NR | Workers: NR  Patients: 40 | Workers: NR  Patients: NR |
| **Civilian and military workers** | | | | |
| Hall et al [36] | March 2020 | 1 | 150  (of which 10 developed symptoms) | 0 |
| de Laval et al [37] | 25 February - 4 March 2020 | NR | 323  (of which 119 at real risk) | 24  (of which 3 asymptomatic) |
| **Travellers** | | | | |
| Draper et al [38] | 1March - 30April2020 | 28 | 445  (of which 80 developed symptoms) | 4 |
| Quach et al [39] | March 2020 | 1 | Primary traced correctly: 183  Secondary: 1000 (quarantined)  Third level: 317 (in self-quarantine) | Primary:15  Secondary: NR  Third level: NR |
| **Vulnerable** | | | | |
| Clarke et al [40] | 6 April - 22 May 2020 | Detainees: 66 probable  (not confirmed)  Prison staff: 45 | Detainees: 5 (+ 9 staff + 25 external)  Prison staff: 448 | Detainees:NR  Prison staff: NR |
| Fields et al [41] | March–May 2020 | PEH: 169 (laboratory test)  GP: 163 (laboratory test) | PEH: 50 identified (of which 31 tested)  24 lost to follow-up in quarantine  GP: 758 identified (of which 322 tested)  0 lost to follow-up in quarantine | PEH: NR          PEH: NR |
