# Supplementary material for: COVID-19 Contact Tracing Strategies During the First Wave of the Pandemic: Systematic Review of Published Studies
Source: JMIR Public Health Surveill. 2023 Jun 23;9:e42678. doi: 10.2196/42678 (PMC10337430; doi:10.2196/42678)
Supplement: Multimedia Appendix 4 [file publichealth_v9i1e42678_app4.docx]

**Multimedia Appendix 4 - Template for Intervention Description and Replication (TIDierR) - Population health and policy interventions (PHP) [24]**

|  | Asiimwe (2021) [25] | Breeher (2020) [32] | Clarke (2020) [40] | de Laval (2021) [37] | Draper (2021) [38] | Fields (2021) [41] | Hall MT (2021) [36] | Kalyanaraman (2021) [26] | Koetter (2021) [27] | Mak (2021) [35] | Mueller (2020) [28] | Niccolai (2020) [29] | Pelton (2021) [30] | Quach (2021) [39] | Reid (2021) [31] | Wong (2020) [34] | Zirbes (2021) [33] | **No. di paper reporting on TIDier-PHP item (X/17)** | **%. di paper reporting on TIDier-PHP item** |
| --- | --- | --- | --- | --- | --- | --- | --- | --- | --- | --- | --- | --- | --- | --- | --- | --- | --- | --- | --- |
| **TIDierR-PHP items [8]** |  |  |  |  |  |  |  |  |  |  |  |  |  |  |  |  |  |  |  |
| 1. Brief name or a phrase that describes the intervention | X | X | X | X | X | X | X | X | X | ? | ? | X | X | X | X | X | X | 15 | 88% |
| 1. Why or the rationale of intervention | X | X | X | X | X | X | X | X | X | X | X | X | X | X | X | X | X | 17 | 100% |
| 1. What - materials | X | X | X | X | X | X | X | X | X | X | X | X | X | X | X | - | X | 16 | 94% |
| 1. What and How - procedure | X | X | X | X | X | X | X | X | X | X | X | X | X | X | X | X | X | 17 | 100% |
| 1. Who provided | X | X | X | - | ? | ? | X | X | X | - | X | X | X | ? | X | - | X | 11 | 65% |
| 1. Where | X | X | X | X | X | X | X | X | X | X | X | X | X | X | X | X | X | 17 | 100% |
| 1. When and how often | X | X | X | X | X | X | X | X | X | X | X | X | X | X | X | X | X | 17 | 100% |
| 1. Planned variation | X | X | - | - | - | - | - | - | - | ? | - | - | - | - | X | - | X | 4 | 24% |
| 1. Unplanned variation | - | - | - | - | - | - | - | - | - | - | - | - | - | - | - | - | ? | 0 | 0 |
| 1. How well | - | - | ? | X | X | X | ? | X | - | ? | X | - | X | X | X | ? | X | 9 | 53% |
| 1. How well - delivery | - | - | - | - | - | - | - | X | - | - | - | - | - | - | - | - | - | 1 | 6% |
| **No. TIDierR-PHP items reported on (X/11):** | 8 | 8 | 7 | 7 | 7 | 7 | 7 | 9 | 7 | 5 | 7 | 7 | 8 | 7 | 9 | 5 | 9 |  |  |
| **% TIDierR-PHP items reported:** | 73% | 73% | 64% | 64% | 64% | 64% | 64% | 82% | 64% | 46% | 64% | 64% | 73% | 64% | 82% | 46% | 82% |  |  |

Legend: X = reported; ? = partially reported; - = not reported.
